# The single flagellum of *Leishmania* has a fixed polarisation of its asymmetric beat

**DOI:** 10.1242/jcs.246637

**Published:** 2020-10-22

**Authors:** Ziyin Wang, Tom Beneke, Eva Gluenz, Richard John Wheeler

**Affiliations:** 1 Sir William Dunn School of Pathology, University of Oxford, Oxford, UK; 2 Peter Medawar Building for Pathogen Research, Nuffield Department of Medicine, University of Oxford, Oxford, UK; 3 The Wellcome Centre for Integrative Parasitology, Institute of Infection, Immunity and Inflammation, University of Glasgow, Glasgow, UK

**Keywords:** *Leishmania*, Flagellum, Cilium, Cell motility, Asymmetry, Trypanosomatid

## Abstract

Eukaryotic flagella undertake different beat types as necessary for different functions; for example, the *Leishmania* parasite flagellum undergoes a symmetric tip-to-base beat for forward swimming and an asymmetric base-to-tip beat to rotate the cell. In multi-ciliated tissues or organisms, the asymmetric beats are coordinated, leading to movement of the cell, organism or surrounding fluid. This coordination involves a polarisation of power stroke direction. Here, we asked whether the asymmetric beat of the single *Leishmania* flagellum also has a fixed polarisation. We developed high frame rate dual-colour fluorescence microscopy to visualise flagellar-associated structures in live swimming cells. This showed that the asymmetric *Leishmania* beat is polarised, with power strokes only occurring in one direction relative to the asymmetric flagellar machinery. Polarisation of bending was retained in deletion mutants whose flagella cannot beat but have a static bend. Furthermore, deletion mutants for proteins required for asymmetric extra-axonemal and rootlet-like flagellum-associated structures also retained normal polarisation. *Leishmania* beat polarisation therefore likely arises from either the nine-fold rotational symmetry of the axoneme structure or is due to differences between the outer doublet decorations.

## Abstract (precis)

**Highlighted Article:** By using high speed, high-resolution fluorescence microscopy of swimming *Leishmania* cells, we showed that the asymmetric flagellar beat always wafts in the same direction and investigate which structures are involved.

## INTRODUCTION

**Fig. 1.**
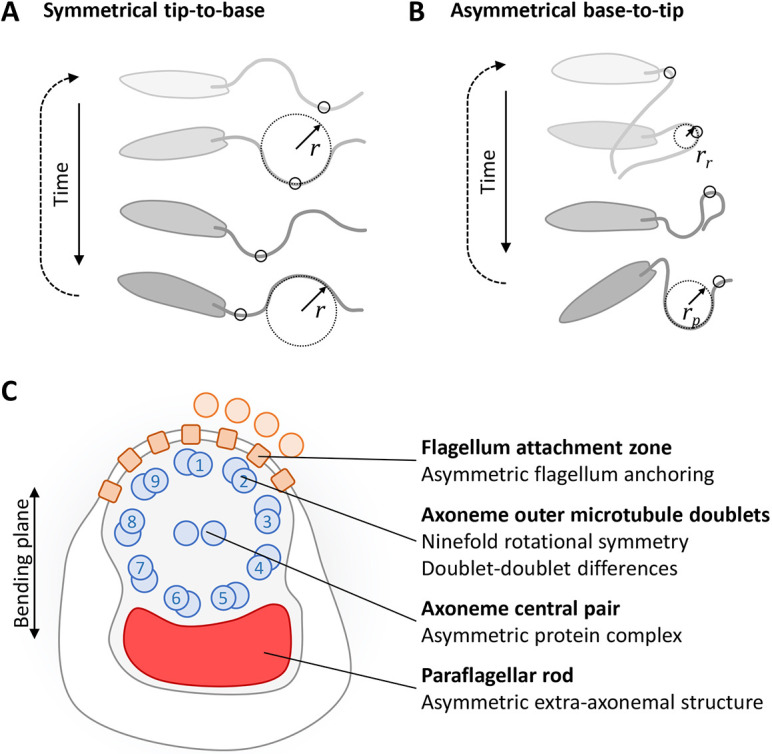
**Known asymmetries in *Leishmania* flagellar beating and flagellum ultrastructure.** (A,B) Schematic representations of the two types of *Leishmania* beat. (A) The symmetric beat, where waves propagate from the tip to the base with symmetric curvature (*r*). (B) The asymmetric beat, where waves propagate from the base to the tip with asymmetric curvature for power and recovery strokes (*r*_p_ and r_r_). (C) Schematic representation of key asymmetries in axoneme structure that may contribute to asymmetry of the base-to-tip beat. This includes asymmetries in the central pair and outer doublets of the axoneme, the PFR and (toward the flagellum base) the attachment to the cell body by the FAZ.

Motile flagella and cilia have essentially indistinguishable ultrastructures but originally received different names based on their biological function – a combination of where they are present in organisms, their structure and the motion they undergo (Takeda and Narita, 2012). The organelle tends to be called a flagellum when they undergo a planar near-symmetric near-sinusoidal beat (Fig. 1A) and there are few per cell. In contrast, the term cilium tends to be used when they undergo a planar strongly asymmetric wafting beat (Fig. 1B) and there are many cilia per cell or many ciliated cells undergoing coordinated movement across a tissue. Ultimately, the correct choice of symmetric or asymmetric waveform, and the correct polarisation of the latter, must be used to achieve the necessary biological function. Defects tend to cause motility defects in swimming cells, and ciliopathies, a range of mild to severe genetic diseases, in humans.

Typical strongly asymmetric beats undergo a power stroke, which drives fluid movement relative to the cell, followed by a recovery stroke, returning the cilium/flagellum to its starting configuration. A planar asymmetric beat has two possible polarisations, corresponding to which way the power stroke pushes fluid as the flagellum beats. For example, in Fig. 1B the flagellum pushes fluid down and rotates the cell anticlockwise while the opposite polarisation would push the fluid up and rotate the cell clockwise. The general assumption seems to be that this polarisation is fixed. In multi-ciliated systems, the polarisation of the power stroke tends to be organised to generate fluid flows [e.g. animal ciliated epithelia, such as in brain ventricles (Faubel et al., 2016)] or to drive cell swimming (e.g. *Tetrahymena* and *Paramecium*) (Naitoh and Kaneko, 1972). There are varied configurations in cells with fewer cilia/flagella; for example, *Chlamydomonas* uses asymmetric beating of two flagella with opposite polarisation to achieve forward swimming (Ringo, 1967), while the unicellular eukaryotic parasite *Leishmania* uses asymmetric beats of its single flagellum to rotate (Gadelha et al., 2007; Holwill and McGregor, 1975) (Fig. 1A,B). Cilia/flagella have asymmetries that contribute to generating the correct beat form – firstly those which keep bending in a plane, and secondly those which introduce asymmetries in the beat with the correct polarisation. Although both structural and functional (e.g. signalling) asymmetries may contribute, far more is known about structural asymmetries.

Motile flagella/cilia canonically have nine outer microtubule doublets in a circular arrangement, around a central pair of singlet microtubules (called the 9+2 arrangement). Their beating is driven by coordinated activity of dynein motors bound to the outer doublet microtubules, which drives sliding between adjacent doublets. To generate planar motion, some structural or regulatory break in this rotational symmetry must exist. Depending on the organism, this may involve addition of a fixed orientation central pair complex (Ishijima et al., 1988), which does not have reflectional or rotational symmetry (Carbajal-González et al., 2013). *Leishmania* and related species have a fixed central pair orientation (Gadelha et al., 2006). Alternatively, it may involve the presence of specialised bridges between particular microtubule doublets to restrict where dynein-driven sliding occurs (Bui et al., 2009; Gibbons, 1961), thus removing the nine-fold rotational symmetry of the outer doublets. Flagella with nine-fold rotational symmetry of the outer doublets and no central pair or asymmetric extra-axonemal structures can undergo three-dimensional rotating or helical movement without a fixed beat plane, such as nodal cilia (Nonaka et al., 1998).

The nature of structural asymmetries that (1) give rise to asymmetric beats and (2) defines their polarisation are less well understood; however, many asymmetries that correlate with asymmetric beat polarisation are known. Within the 9+2 axoneme, differences between the outer doublet decoration, particularly the inner dynein arms, are likely important (Bui et al., 2009, 2012). In addition to the asymmetric 9+2 axoneme, cilium-associated structures tend to be asymmetric. This includes the anchoring of the basal body to the cell by rootlet structures – such as the basal body–basal body linkage mediated by the distal striated fibre in *Chlamydomonas* (Dutcher and O'Toole, 2016; Ringo, 1967), the basal foot structure in ciliated animal epithelia cells (Gibbons, 1961) and the basal body-associated structures in *Paramecium* (Tassin et al., 2016). Mutations of rootlet proteins cause a loss of ciliated tissue polarity. However, individual cilia rotate randomly while retaining their normal asymmetric structure (Clare et al., 2014; Kunimoto et al., 2012). Each individual cilium presumably retains their asymmetric beat, although this has not been analysed in detail.

An asymmetric beat can arise when a flagellum has a large static curvature (the underlying ‘shape’ of the flagellum) in addition to the symmetric dynamic curvature (the propagating wave) (Eshel and Brokaw, 1987; Geyer et al., 2016). In *Chlamydomonas*, several mutants that have a more symmetric beat are known, including *pf2* (Brokaw and Kamiya, 1987) and *mbo2* (Segal et al., 1984). Generation of static and dynamic curvature are separable, and *mbo2* has a greatly reduced static curvature (Geyer et al., 2016). However, no known mutants invert the bend of this static component to invert the polarisation of the beat.

It is unknown whether a single *Leishmania* flagellum has a fixed polarisation for its asymmetric beat. We therefore asked whether polarisation is fixed or switchable, and what structures could be involved in conferring asymmetry (Fig. 1C). In addition to the 9+2 axoneme, *Leishmania* have an extra-axonemal structure called the paraflagellar rod (PFR). There are several possible functions of the PFR (Portman and Gull, 2010), which is required for normal flagellum beating (Santrich et al., 1997). Its protein composition includes proteins with Ca^2+^- and cAMP-associated functions which may regulate beating (Oberholzer et al., 2007; Portman et al., 2009), while its structure suggests biomechanical effects to convert planar into three dimensional movement (Hughes et al., 2012; Koyfman et al., 2011), although we have previously argued against the latter (Wheeler, 2017). The PFR has a fixed asymmetric position and is attached to outer microtubule doublets 4–6 (Fuge, 1969; Gadelha et al., 2005). The anchoring of the flagellum base to the cell is also asymmetric, analogous to rootlet structures in other organisms, with lateral attachment to the cell body in the flagellar pocket via the flagellum attachment zone (FAZ) only near outer microtubule doublets 9, 1 and 2 (Wheeler et al., 2016). The 9+2 axoneme itself likely also has asymmetries between outer microtubule doublets, based on recent cryo-electron tomography of the related species *Trypanosoma brucei* (Imhof et al., 2019)*.*

Analysing polarisation of *Leishmania* asymmetric beats has a key challenge – *Leishmania* cells appear axially symmetric when viewed though transmitted light illumination. To determine the cellular orientation in swimming cells, we developed a high frame rate dual-colour widefield epifluorescence technique for visualising *Leishmania* promastigotes, allowing us to observe the asymmetric internal cytoskeletal structure (labelled with a fluorescent protein) while also observing flagellum beating as the cell undergoes different flagellum beat behaviours. By combining this with mutations in different asymmetric features of the flagellum and cell–flagellum attachment, we showed (1) that the flagellum has a fixed polarisation of the asymmetric flagellar beat, (2) paralysed flagellum mutants that form a static curvature retain a fixed polarisation, and (3) this asymmetry does not require the large PFR structure or lateral attachment through the FAZ. This has implications for the mechanisms by which this parasite may achieve directed taxis.

## RESULTS

**Fig. 2.**
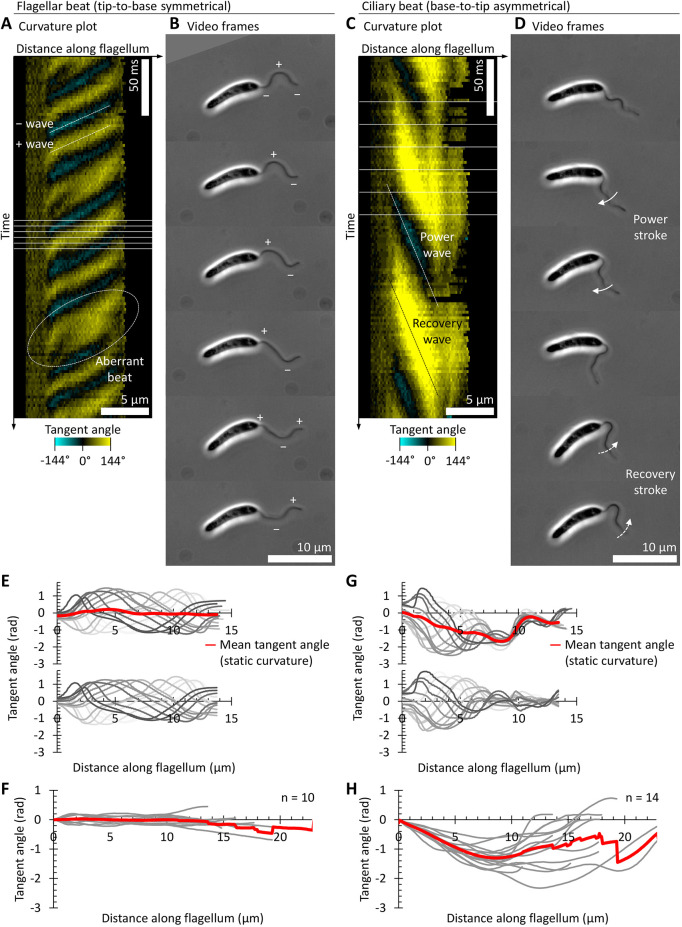
**The symmetric and asymmetric beats of the *Leishmania* flagellum.** (A–D) One example cell undergoing either (A,B) a symmetric tip-to-base flagellar beat or (C,D) an asymmetric base-to-tip ciliary beat visualised from a 400 Hz high-frame rate phase-contrast video. (A) The symmetric beat, plotted as a kymograph of tangent angle along the flagellum, automatically traced from the phase-contrast video. Yellow indicates clockwise tangent angles relative to the flagellum base, and cyan anticlockwise tangent angles. The waves during the symmetric beat propagate from the flagellum tip to its base (from top right to bottom left), alternating between waves with a negative tangent angle (labelled ‘−’) or a positive tangent angle (labelled ‘+’). Aberrant waves occasionally occur with a different frequency or with additional bends in the flagellum; one example is indicated. (B) Six frames illustrating the propagation of positive and negative tangent angle waves over one beat cycle. Phase-contrast images from the frames indicated with horizontal lines in A. (C) Kymograph of tangent angle for the asymmetric beat. The waves propagate from the flagellum base to its tip (from top left to bottom right), alternating between a wave for a power stroke (small tangent angles, negative in this example) and a wave for a recovery stroke (large tangent angles, positive in this example). (D) Six frames illustrating the power and recovery strokes over one beat cycle as indicated in C. (E) Top, plotted tangent angles at different distances along the flagellum for one symmetric tip-to-base beat cycle from the experiment in A,B. The mean tangent angle at each distance along the flagellum gives the static curvature of the flagellum, represented as the tangent angle, which is very near straight. Bottom, tangent angles with static curvature subtracted. (F) Average static curvature for many flagella undergoing tip-to-base beats (grey), and their mean. (G) Tangent angle plotted as in E, except for one asymmetric base-to-tip beat cycle from the experiment in C,D. (H) Average static curvature for many flagella undergoing base-to-tip beats (grey), and their mean.

*Leishmania* flagellum beating can be readily analysed from high frame rate (400 Hz) phase-contrast videos using automated tracing of the flagellum configuration (Walker and Wheeler, 2019; Walker et al., 2019). As often used for other organisms, this data can be represented as plots of tangent angle at different distances along the flagellum over time (Fig. 2; Movie 1), where tangent angle is the angle of a section of flagellum relative to the flagellum base. A higher rate of change in tangent angle along the flagellum corresponds to higher flagellum curvature. *Leishmania* undergo two well-described types of flagellum beating, a high frequency tip-to-base beat and a lower frequency base-to-tip beat. These occur at ∼20 to 25 Hz for the tip-to-base and ∼5 Hz for the base-to-tip beats (Gadelha et al., 2007; Wheeler, 2017). The tip-to-base beat is symmetric (typical of flagella), while the base-to-tip beat is asymmetric with a power and recovery stroke (typical of cilia) (Gadelha et al., 2007; Holwill and McGregor, 1975).

In tangent angle plots, the wavefronts of the high frequency tip-to-base symmetric beat appear as lines aligned top right to bottom left, alternately with positive or negative tangent angles (Fig. 2A; Movie 1A). These correspond to the successive wavefronts propagating from the flagellum tip over time (Fig. 2B). The wavefronts of the asymmetric base-to-tip beat appear as wider diagonal lines aligned top left to bottom right, corresponding to lower frequency waves propagating from the flagellum base over time (Fig. 2C; Movie 1B). Unlike the symmetric beat, the alternating positive and negative tangent angle wavefronts in the asymmetric beat have different magnitudes of tangent angle (Fig. 2C,D).

The conformation of the flagellum can be mathematically separated into two components – a moving component (dynamic curvature) and a static component (static curvature). The tangent angles arising from the static curvature can be determined by averaging the tangent angle over an integer number of beats or by taking the static/infinite frequency mode of a Fourier decomposition. In *Chlamydomonas*, it has been shown that generation of dynamic and static curvature are biologically separable and not just mathematical constructs (Geyer et al., 2016). While undergoing a symmetric tip-to-base beat the *Leishmania* flagellum static curvature is low (Fig. 2E,F). In contrast, for asymmetric base-to-tip beats, the tangent angle reaches large values (>*π*/2 rad, >90°) corresponding to a high static curvature (Fig. 2G,H). Dynamic components of flagellum conformation can be determined by subtracting the static curvature. This corresponds to the propagating wave shape on the underlying static shape of the flagellum. Tangent angles arising from dynamic curvature of both the tip-to-base and base-to-tip beats are near symmetric; however, the base-to-tip beats often do not propagate along the entire flagellum (Fig. 2G). For the proximal portion of the flagellum, the tangent angle has a near-linear correlation with distance along the flagellum, corresponding to a constant curvature (Fig. 2H), similar to what is observed in the whole flagellum asymmetric in *Chlamydomonas* beats (Geyer et al., 2016).

### The asymmetric *Leishmania* beat occurs with a constant polarisation

**Fig. 3.**
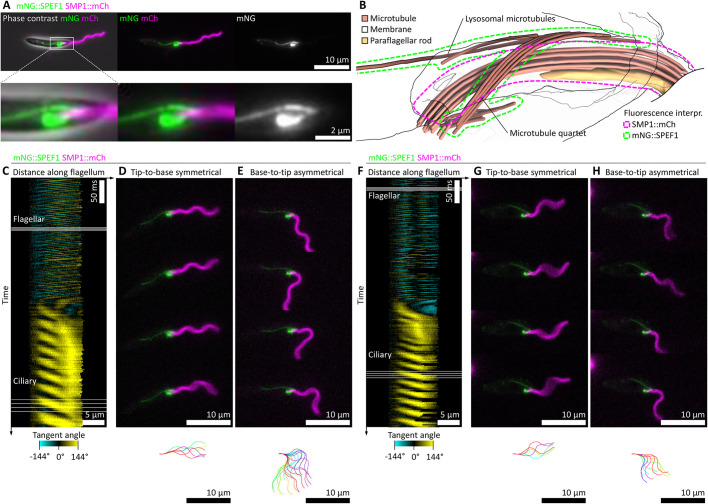
**SPEF1 is an asymmetric reporter of *Leishmania* cell orientation during asymmetric flagellum beating.** (A) The subcellular localisation of the microtubule quartet and lysosomal microtubule-associated mNG::SPEF1 relative to the flagellar membrane marker SMP1::mCh as determined by epifluorescence microscopy. (B) Structure of the flagellar base and flagellar pocket as determined by electron tomography, as previously published (Wheeler et al., 2016), indicating the SPEF1- and SMP1-associated ultrastructures. (C–H) Two examples of flagellum movement and cell orientation in cells expressing mNG::SPEF1 and SMP1::mCh switching from a symmetric tip-to-base to an asymmetric base-top-tip beat, derived from 100 Hz high frame rate dual-colour epifluorescence videos. (C–E) First example cell. (F–H) Second example cell. (C,F) Change in tangent angle over time, automatically traced from the SMP1::mCh signal. Yellow indicates tangent angles to the right, cyan to the left. (D,G) Four frames showing the symmetric tip-to-base beat. mNG::SPEF1 and SMP1::mCh fluorescence from the frames indicated “Flagellar” in C,F. The flagellum configuration over one beat cycle is shown at the bottom. (E,H) Four frames showing the asymmetric base-to-tip beat. mNG::SPEF1 and SMP1::mCh fluorescence from the frames indicated “Ciliary” in C,F. The asymmetric beat tends to bend away from the side of the cell with the lysosomal microtubule.

*Leishmania* have a fixed axoneme central pair orientation (Fig. 1C), and bending for flagellum beating is thought to occur only in the plane perpendicular to the central pair (Gadelha et al., 2006). Therefore, there are two possible directions for the power stroke of the asymmetric beat. Ciliary beating in other organisms is highly polarised, suggesting a single preferred direction; however, the *Leishmania* cell is near-axially symmetric. The cell body can be slightly curved; however, not every cell is curved and it is not known whether this curvature occurs in a consistent direction relative to flagellar structures. Therefore, the orientation of flagellar structures, and thus polarisation, of the asymmetric beat cannot be inferred from transmitted light images such as phase-contrast images (Fig. 2). Whether there is a preferred direction can be tested by observing asymmetric intracellular structures, which act as a reporter of cell orientation, during flagellum beating. We achieved this using high frame rate dual-colour widefield epifluorescence microscopy (Fig. S1) using a cell line expressing the well-characterised flagellum membrane marker SMP1::mCh (Tull et al., 2004; Wheeler et al., 2015) and a marker of the asymmetric microtubule-based cytoskeletal structure, comprising the microtubule quartet and the lysosomal microtubule(s), mNG::SPEF1 (Gheiratmand et al., 2013; Halliday et al., 2018; Wang et al., 2019) (Fig. 3A).

Through comparison to previously published electron tomography of the flagellar pocket (Wheeler et al., 2016), the relative orientation of the flagellum axoneme, PFR and FAZ can be inferred from the appearance of the mNG::SPEF1 signal (Fig. 3B). In Fig. 3B, the plane of beating is the plane of the image (perpendicular to the central pair). As the flagellum exits the cell through the flagellar pocket neck, it attaches to the microtubule quartet via the FAZ on one side of the flagellum. This attachment region is near where specialised lysosome-associated microtubule(s) also meet the microtubule quartet, on the opposite side of the flagellum to the start of the PFR (Wheeler et al., 2016).

In high frame rate videos, the flagellum movement can be visualised and traced from the SMP1::mCh signal. Identifying example cells where the beat switches from symmetric to asymmetric over the course of the video is relatively easy, illustrated for two example cells in Fig. 3C–H and Movie 2. The switch between flagellum beats is summarised by tangent angle at different distances along the flagellum over time (Fig. 3C,F). In these videos, the orientation of bending relative to the cellular ultrastructure can be inferred from the mNG::SPEF1 signal, both while undergoing a symmetric beat (example frames and the traced beat shown in Fig. 3D,G) or an asymmetric beat (Fig. 3E,H). This showed that the power stroke of the asymmetric beat pushed away from the side of the cell with the lysosomal microtubule(s), corresponding to a static curvature toward that side of the cell.

We noted that the symmetric to asymmetric beat switching involved the following stages. First, a tip-to-base wave that stalls and then reverses, giving a bend that gradually propagates towards the flagellum tip. Second, high frequency tip-to-base waves that continue to initiate near the flagellum tip but fail to propagate past the stalled/reversed bend. Third, lower frequency asymmetric base-to-tip waves that start to initiate at the flagellum base but fail to propagate past the stalled/reversed bend. This can give both base-to-tip waves in the proximal domain and tip-to-base waves in the distal domain of a single flagellum at the same time (which is unlike previously described (Gadelha et al., 2007)). Finally, base-to-tip waves that can propagate along the whole flagellum and with no new tip-to-base waves initiated. Initiation of new tip-to-base waves can stop after after the first base-to-tip wave but may take several base-to-tip waves. The gradually propagating bend in the first stage is likely associated with a base-to-tip establishment of the static curvature for the asymmetric beat.

### The PFR is on the inside of the tightly curved recovery stroke

**Fig. 4.**
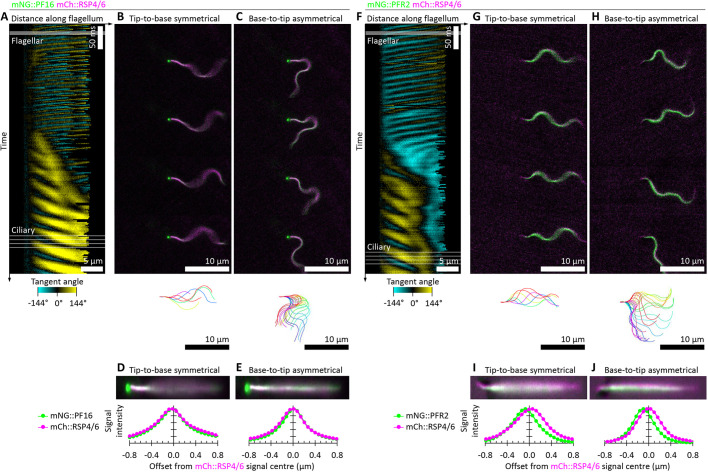
**The position of the asymmetric PFR extra-axonemal structure in beating flagella.** (A–E) Flagellum movement in a cell expressing mNG::PF16 and mCh::RSP4/6 (two axonemal proteins – central pair and radial spokes, respectively) switching from a symmetric tip-to-base to an asymmetric base-top-tip beat, derived from a 100 Hz high frame rate dual-colour epifluorescence video. (A) Change in tangent angle over time, as in Fig. 3C, automatically traced from the mNG::PF16 signal. (B) Four frames showing the symmetric tip-to-base beat and the flagellum configuration over the beat cycle. (C) Four frames showing the asymmetric base-to-tip beat and the flagellum configuration over the beat cycle. (D) Digitally straightened mNG::PF16 and mCh::RSP4/6 signal, averaged over 64 frames of tip-to-base symmetric beating, presented stretched in the transverse direction by 2**×**, and the transverse signal intensity profile. (E) As for D, but for 64 frames of tip-to-base asymmetric beating. There is no offset between mNG::PF16 and mCh::RSP4/6 signal. (F–J) As for A–E, except a cell expressing mNG::PFR2 and mCh::RSP4/6 (a PFR and an axonemal protein, respectively). The mNG::PFR2 signal is offset from mCh::RSP4/6 signal. During asymmetric beating, the flagellum curves with the PFR on the inside of the tighter curvature recovery stroke.

The analysis of cell asymmetry using mNG::SPEF1 (Fig. 3C–H) in combination with the ultrastructure of the cell (Fig. 3B) indicates that the PFR sits on the leading side of the flagellum during the asymmetric beat power stroke, which corresponds to the inside of the static curvature. To directly confirm this result, we analysed two cell lines, one expressing two axoneme markers (mNG::PF16 and mCh::RSP4/6), and one expressing a PFR marker and an axoneme marker (mNG::PFR2 and mCh::RSP4/6). In the former, the red and green signal should precisely colocalise, while in the latter there should be a small offset – ∼150 nm based on flagellum ultrastructure (Fig. 1C). Again, it was relatively easy to find examples of cells where the beat switches from symmetric to asymmetric (Fig. 4; Movie 3). The mCh::RSP4/6 signal was much weaker than SMP1::mCh; therefore, to analyse these videos, we used automated tracing to determine the configuration of the flagellum, then digitally straightened the flagellum from each frame of the video. Multiple frames can then be averaged, to increase signal relative to background noise, allowing precise measurement of red–green signal offset. This showed that there was a precise colocalisation of the two axoneme markers (mNG::PF16 and mCh::RSP4/6; Fig. 4A–E; Movie 3A), both in straightened images from symmetric beats (Fig. 4D) and asymmetric beats (Fig. 4E). In contrast, the PFR was consistently offset from the axoneme (mNG::PFR2 and mCh::RSP4/6; Fig. 4F–J; Movie 3B). The direction of the offset indicates the PFR is on the inside of the static curvature, and the offset was consistent with the expected position of the PFR relative to the axoneme.

**Fig. 5.**
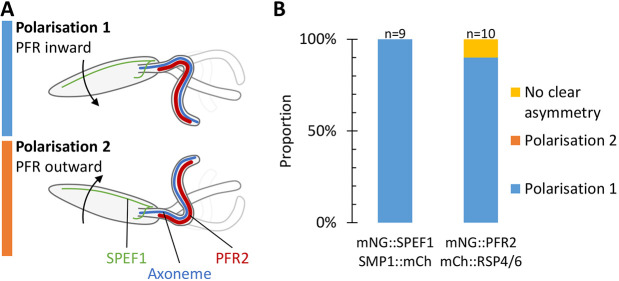
**Asymmetric base-to-tip beating occurs in a consistent orientation.** (A) *Leishmania* flagellum beating is near-planar, giving two possible polarisations for the asymmetric base-to-tip flagellum beat. Either polarisation (1), with the PFR on the inside of the narrow radius recovery stroke bend, or (2), with the PFR on the outside of the recovery stroke bend. (B) Instances of polarisation 1 or 2, counted from high frame rate fluorescence videos of cells expressing mNG::SPEF1 and SMP1::mCh (as in Fig. 3) or mNG::PFR2 and mCh::RSP4/6 (as in Fig. 4F–J). The asymmetric beat has a strong preference for the power stroke to bend away from the side of the cell with the lysosomal microtubule, positioning the PFR on the inside of the tightly curved recovery stroke.

By using many high frame rate dual-colour widefield epifluorescence microscopy videos of either the mNG::SPEF1/SMP1::mCh (*n*=9) or mNG::PFR/mCh::RSP4/6 (*n*=10) cell lines, we could determine the incidence of the two possible polarisations of the asymmetric beat – either with the power stroke bending away from the side of the cell with the lysosomal microtubules with the PFR sitting on the leading side of the flagellum or the inverse (Fig. 5A). The results indicate that there is a strong and consistent polarisation of the asymmetric beat (Fig. 5B), with a tighter radius of curvature of the reverse bend (doublet 1 on the outside of the curve) than the principal bend (doublet 1 on the inside). This indicates that the PFR experiences greater compression during the recovery stroke/reverse bend than extension during the power stroke/principal bend.

To determine how the asymmetric structures of the *Leishmania* flagellum and associated structures (Fig. 1C) contribute to this asymmetric behaviour, we analysed flagellum bending in mutants of the axoneme components required for motility, PFR and the FAZ.

### Mutants only able to form a static bend have inverted polarisation to asymmetric beats

**Fig. 6.**
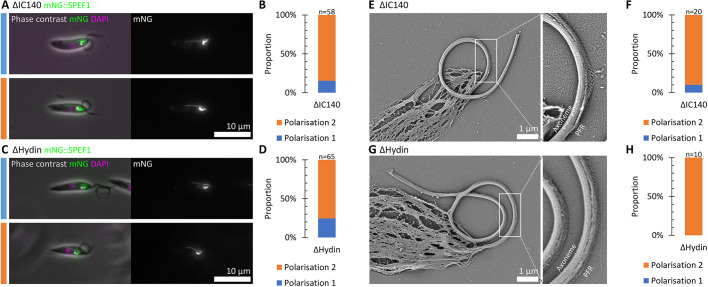
**Axoneme protein deletion mutants with a paralysed ‘curled flagellum’ phenotype have a preferred curl direction.** (A) Example fluorescence micrographs of a IC140, an inner dynein arm intermediate chain, deletion mutant (ΔIC140) expressing mNG::SPEF1, with the flagellum either curling toward the side of the cell with the lysosome microtubule (polarisation 1, PFR on the outside of the curve) or the opposite (polarisation 2). (B) Proportion of cells, assessed from light microscopy, with the flagellum curling with polarisation 1 or 2. (C,D) As for A,B, except for a deletion mutant of Hydin (ΔHydin), a central pair complex protein. (E) Example scanning electron micrograph of a detergent-extracted cytoskeleton from an IC140 deletion mutant cell. The direction of curling is directly visible from the PFR next to the axoneme. (F) Proportion of cells, assessed from SEM, with the flagellum curling with polarisation 1 or 2. (G,H) As for E–F, except for a deletion mutant of Hydin. These mutants with dysregulation of flagellum beating still have a preferred bend direction, albeit for a non-beating flagellum.

In *Leishmania*, deletion mutants for many conserved axoneme proteins have a paralysed flagellum unable to undergo a beat. However, in a subset of deletion mutants, the paralysed flagella retain some capacity for bending, and a large proportion have a curled configuration – typically a few turns of a coil (Beneke et al., 2019). These mutants are not informative for understanding waveform generation; however, they form a strong static curvature. We tested whether this static curvature retained a preferred polarisation using two mutants where curling is highly prevalent (Beneke et al., 2019) to determine whether polarised bending can occur in the absence of flagellar beating. The first was a deletion of the inner dynein arm intermediate chain protein IC140 (Hendrickson et al., 2013; Heuser et al., 2012). The second was a deletion of Hydin, a central pair complex protein required for central pair microtubule stability and to prevent central pair rotation (Dawe et al., 2007). Again, we used mNG::SPEF1 as a reporter to allow determination of the direction of flagellum bending. Curled flagella in both the ΔIC140 and ΔHydin mutant remained in the normal beat plane and had a preferred bend direction towards the side of the cell with the lysosomal microtubule. This corresponds to the PFR lying on the outside of the coil, although curling in both directions did occur (Fig. 6A–D). To confirm this result, we used scanning electron microscopy (SEM) of detergent-extracted cytoskeletons of these two mutants. This allowed direct visualisation of the PFR on the outside face of a large majority of coiled flagella (Fig. 6E–H).

These curled flagella therefore have a strong static curvature in the opposite direction to the static curvature of the asymmetric base-to-tip beat. These flagella were strongly curved – the tangent angle at the flagellum tip could reach >2*π* rad, >360° (Fig. 6A,C), with a tight curvature radius of 2.70±0.42 μm and 2.61±0.21 μm (mean±s.d.) for ΔHydin and ΔIC140, respectively. Interpreting this result is difficult as it is not clear that the flagellum curling direction in these mutants originates from the same molecular mechanism as the asymmetry of the normal asymmetric beat. However, it is clear that upon disruption of either the inner dynein arms (ΔIC140) or the central pair (ΔHydin), the flagellum retains the ability to have polarised static curvature while losing the ability for dynamic curvature owing to dysfunction within the 9+2 axoneme.

### Disruption of asymmetric extra-axonemal flagellum structures does not alter polarisation

**Fig. 7.**
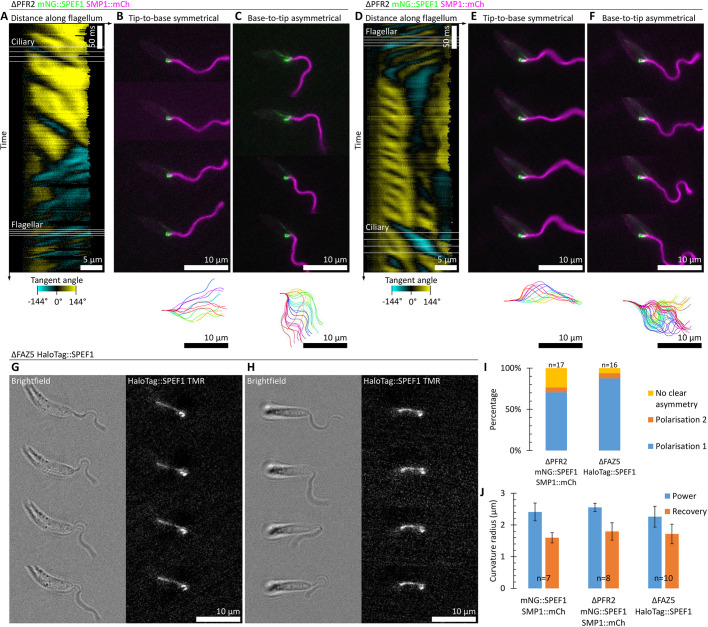
**Neither disruption of the PFR nor the FAZ inverts asymmetric beat polarity.** (A–F) Flagellum movement in two example cells of a PFR2 (a PFR protein) deletion mutant (ΔPFR2) expressing mNG::SPEF1 and SMP1::mCh switching from a symmetric tip-to-base to an asymmetric base-to-tip beat, derived from a 100 Hz high frame rate dual-colour epifluorescence video. (A) Change in tangent angle over time, as in Fig. 3C, automatically traced from mNG::PF16 signal. (B) Four frames showing the symmetric tip-to-base beat and the flagellum configuration over the beat cycle. (C) Four frames showing the asymmetric base-to-tip beat and the flagellum configuration over the beat cycle. (D–F) As for A–C except for the second example cell with a base-to-tip beat with no clear asymmetry. (G,H) Flagellum movement in two example cells of a FAZ5, a FAZ protein, deletion mutant (ΔFAZ5) expressing HaloTag::SPEF1 and labelled with Tetramethylrhodamine ligand. Both examples persistently underwent an asymmetric base-to-tip beat, summarised with four frames from a 100 Hz high frame rate green light brightfield and red fluorescence video. (I) Instances of asymmetric bending in the direction 1 or 2 (as defined in Fig. 5), counted from high frame rate fluorescence videos of either PFR2 or FAZ5 deletion mutants. The asymmetric beat retains a strong preference for the power stroke to bend away from the side of the cell with the lysosomal microtubule, with more examples of reverse beats with unclear asymmetry in the PFR2 deletion. (J) Curvature of the power and recovery strokes measured from fluorescence videos of PFR2 or FAZ5 deletion mutants relative to a cell line with no deletion.

The PFR is a large extra-axonemal structure of comparable size to the axoneme. It is specific to the euglenid lineage of unicellular eukaryotes and is asymmetrically positioned in the flagellum next to doublets 4 to 6 of the axoneme (Fig. 5C). PFR2 is a major structural component of the PFR and deletion of PFR2 leads to loss of almost all of the PFR. In *Leishmania* this leads to a flagellar beat that is still dominantly tip-to-base but with a shorter wavelength and lower amplitude, leading to slower forward swimming (Santrich et al., 1997). A similar motility defect also occurs upon PFR2 deletion in the related parasite *Trypanosoma brucei* (Bastin et al., 1999), where it was also shown that PFR2 deletion does not affect the fixed central pair orientation (Gadelha et al., 2006). To determine whether loss of the PFR alters the base-to-tip asymmetric beat, we generated a PFR2 deletion cell line expressing mNG::SPEF1 and SMP1::mCh (Fig. 7; Movie 4). Plots of tangent angle over time show the multiple defects the flagellum beat experiences in the absence of the PFR (Fig. 7A,D). As previously described, flagella still predominantly undergo tip-to-base waveforms, but examples of base-to-tip waveforms were also readily identifiable. Tangent plots confirm that bending tends to be lower amplitude and many wavefronts fail to propagate along the entire length of the flagellum. The frequency was also variable. The lower amplitude flagellum movement also allows the cell to rotate more readily between the slide and coverslip, complicating analysis. Nonetheless, the tip-to-base beat still tends to be near-symmetrical, if a little uncoordinated, and in the normal beat plane (Fig. 7B,E). The more infrequent base-to-tip beat is still often asymmetric with normal polarity and in the normal beat plane (Fig. 7C); however, in comparison to the parental line (Figs 3 and 5), there were also more cells with less pronounced asymmetry (Fig. 7F,I).

Finally, we considered the asymmetrically positioned FAZ at the base of the flagellum. Previous work has identified the protein FAZ5 as vital for any lateral attachment between the flagellum and the flagellar pocket neck, in turn giving rise to a motility defect (Sunter et al., 2019). To determine whether this defect was associated with altered asymmetry of the base-to-tip beat, we generated a FAZ5 deletion cell line expressing HaloTag::SPEF1, where HaloTag is a self-labelling protein tag which covalently binds a chloroalkane with an amine-linked fluorophore – in this case Tetramethylrhodamine. HaloTag was used as the ΔFAZ5 cell line was, for an unknown reason, refractory to tagging of SPEF1 with conventional β-barrel fluorophores (mNG, eYFP and mCh). We were also unsuccessful at generating a ΔFAZ5 cell line using a single selectable marker, leaving insufficient selectable markers to also tag SMP1. Therefore the cell line was visualised using a combination of red fluorescence and bright field transmitted light using a green filter for simultaneous visualisation of the flagellum and the orientation of the cell using HaloTag::SPEF1 (Fig. 7G,H; Movie 5). Flagella tended to undergo a base-to-tip beat, suggesting a reduced ability for a tip-to-base beat is the origin of the previously observed swimming defect (Sunter et al., 2019). Intriguingly, this suggests the FAZ represses the base-to-tip beat. The bright-field videos had insufficient contrast for automated tracing of the flagellum beat, but the base-to-tip beats appeared normal, consistently occurred with normal polarisation and consistently occurred in the normal beat plane (Fig. 7G,H; Movie 5). The combined evidence from the ΔPFR2 (*n*=17) and ΔFAZ5 (*n*=16) mutants suggest that the most prominent asymmetric flagellum-associated structures in *Leishmania* are not required for achieving asymmetric static flagellum curvatures (Fig. 7I, cf. Figs 3 and 5) and do not affect the curvature of the power and recovery strokes (Fig. 7J).

## DISCUSSION

*Leishmania* are highly genetically tractable cells (Beneke et al., 2017) and the promastigote life cycle stages have a canonical 9+2 single flagellum that switches between a near-planar (Walker and Wheeler, 2019) symmetric tip-to-base beat and an asymmetric base-to-tip (Gadelha et al., 2007; Holwill and McGregor, 1975), making it an excellent system for understanding the fundamental biology of flagella/cilia and control of their flagellar beating. In particular, new opportunities to analyse the origin and switching of beat asymmetry arise as the symmetric beat propagates from tip-to-base and the asymmetric beat from base-to-tip.

*Leishmania* have a near-axially symmetric cell shape, which makes it challenging to analyse cell orientation from transmitted light micrographs. To overcome this limitation, we developed the first dual-colour high resolution and high frame rate fluorescence visualisation of live swimming cells and used this to visualise proteins endogenously tagged with genetically encoded fluorophores (mCh or mNG) or fluorophore-binding proteins (HaloTag), through either simultaneous capture of red and green fluorescence, or red fluorescence along with green transmitted light. This allowed us to analyse polarisation of flagellum beating relative to the orientation of flagellum and flagellum-associated intracellular ultrastructure (Fig. 3), despite the outward axially symmetric appearance of *Leishmania.*

Our analysis showed that the asymmetric *Leishmania* beat has a fixed polarisation (Fig. 5) that could be mathematically described as a fixed direction for static curvature (Fig. 3). The fixed polarisation means the PFR and microtubule doublets 4 and 5 always lie on the inside of the static curvature and the inside of tightly-curved power strokes. As *Leishmania* is a very early-diverging eukaryote, it is likely that that a fixed polarisation of strongly asymmetric beats is universal among eukaryotes, building on the lack of evidence for polarisation switching in other systems. For example, Ca^2+^-mediated regulation of ciliated epithelia beating involves an arrest in beating or change in frequency rather than switching polarisation and Ca^2+^-mediated regulation of *C. reinhardtii* beating involves a switch from an asymmetrical to a symmetrical beat rather than switching polarisation (Inaba, 2015). The only possible example of a switch in asymmetric beat polarisation we identified is the ‘ciliary reversal’ described for *Paramecium* (Eckert, 1972; Naitoh and Kaneko, 1972). However, the *C. reinhardtii* switch to symmetrical movement is also confusingly described as ‘flagellar reversal’ (Schmidt and Eckert, 1976) despite not involving reversal of either waveform direction or polarity, leaving the true nature of the waveform change in *Paramecium* ciliary reversal unclear.

Rheotaxis and chemotaxis are complex behaviours with many contributing mechanisms; however, a fixed polarisation of asymmetric beat may restrict the possible responses *Leishmania* can undergo to direct its swimming. This can be illustrated with a hypothetical 2D situation: on approaching and sensing a barrier at an oblique angle *Leishmania* could not chose to turn away from the wall by switching to an asymmetric beat, only to turn in the direction the fixed polarisation asymmetric beat allows. *Leishmania* can undergo directed taxis (osmotaxis) (Leslie et al., 2002); however, how flagellum movement is modulated to do so is not yet clear. If we assume that the only mechanism is the switch from the symmetrical tip-to-base beat to the asymmetric base-to-tip beat, then the cell cannot simply turn to align itself with the osmotic gradient making a run-and-tumble-like mechanism, with tumbles caused by the base-to-tip beat, most plausible. However, our results do not preclude other modulations of the flagellar beat to achieve directed taxis. Sperm of many species have small asymmetries in the flagellum beat leading to circular or helical swimming paths. By modulating this asymmetry, periodic changes to the curvature of the swimming path can give rise to robust chemotaxis (Kaupp et al., 2008). Making detailed comparisons between chemotaxis/rheotaxis mechanisms in sperm (a head first ‘pusher’) in *Leishmania* (a ‘puller’) may not be accurate. However, it is notable that sperm trajectories tend to constantly turn in the same direction and it seems plausible that the small asymmetries in sperm flagella may also have a fixed polarisation.

In *Chlamydomonas*, regulation of the symmetric dynamic curvature of the flagellum to make a wave propagate and the static curvature of the flagellum to introduce asymmetry are biologically separable – mutant (demonstrated with *mbo2*) flagellum can fail to form a static curvature giving an aberrant symmetric waveform but otherwise normal flagellum beating (Brokaw and Kamiya, 1987; Geyer et al., 2016; Segal et al., 1984). In *Leishmania*, no mutants that lose the asymmetry of their base-to-tip beat are yet known – deletions of the outer dynein arm-associated proteins dDC2 and LC4-like do promote asymmetric or symmetric beats, respectively, but these beats occur in their normal respective base-to-tip and tip-to-base propagation directions (Edwards et al., 2018). However, there are *Leishmania* deletion mutants (including PF16, Hydin and IC140) where the flagellum is paralysed but often curls up (Beneke et al., 2019) while, naïvely, paralysed flagella would be expected to be straight. Deletions of IC140 and Hydin are the most dramatic examples. The curling in these mutants is still polarised (Fig. 6); however, this polarisation is in the opposite direction to the static curvature in base-to-tip asymmetric beats (Fig. 5). This strongly suggests that flagellar polarisation is retained in the absence of the central pair complex and in the absence of the IC140-associated inner dynein arms, although the precise correspondence of this mutant phenotype to normal symmetric or asymmetric beats is unclear.

*Leishmania* has additional asymmetric flagellum-associated structures that may have been important for conferring flagellum beat polarisation. However, loss of lateral flagellum attachment by the FAZ did not reduce asymmetry or invert the polarisation of the asymmetric base-to-tip beat (Fig. 7). Loss of almost the entire bulk of the PFR also did not invert the polarisation of the asymmetric base-to-tip beat (Fig. 7). The FAZ is specific to the *Leishmania* branch of life, but asymmetric rootlet structures analogous to the FAZ are often present in other organisms. Similarly, flagella in other species can have extensive extra-axonemal structures analogous to the PFR, such as the outer dense fibres and thickened fibrous sheath regions of metazoa sperm. Together, this suggests that asymmetric extra-axonemal structures and rootlet structures in other species are not likely to be responsible for the polarisation of asymmetric flagellum beats. Both the PFR2 and FAZ5 deletion mutants did, however, perturb the flagellum beat in other ways. Interpreting these beat defects is unfortunately complex as each deletion leads to disruption of a large complex cytoskeletal structure with many (likely >100) components (Portman and Gull, 2010; Sunter and Gull, 2016; Sunter et al., 2019).

Taken together, this work greatly constrains the molecular origin of asymmetry in *Leishmania* base-to-tip beats. Our previous work indicated that outer dynein arm-associated factors are likely important for switching between symmetric tip-to-base and asymmetric base-to-tip beats (Edwards et al., 2018); however, controlling switching is distinct from the actual generation of asymmetry. Having clearly excluded the FAZ and PFR, and provided some evidence against the axoneme central pair and IC140-containing inner dynein arms being responsible for asymmetry, the most likely remaining candidate is differences between the outer doublet decorations – in particular in the region of inner arm dynein b, based on cryo-electron tomography of *T. brucei* (Imhof et al., 2019). However, the mechanical properties of the nine-fold asymmetric outer microtubule doublets of the axoneme itself could also be important. Specialised inner dynein arm are also implicated in *Chlamydomonas* flagellum movement asymmetries (Bui et al., 2009), perhaps indicating that this is the eukaryote-wide origin for asymmetry of flagellum movement.

## MATERIALS AND METHODS

Procyclic promastigote *L. mexicana* expressing Cas9 and T7 polymerase, derived from WHO strain MNYC/BZ/62/M379 (Beneke et al., 2017) were grown in M199 medium with Earle's salts and L-glutamine (Life Technologies) supplemented with 26 mM NaHCO_3_, 5 μg ml^−1^ haemin, 40 mM, HEPES-NaOH (pH 7.4) and 10% fetal calf serum (FCS). *L. mexicana* were grown at 28°C and maintained at culture densities between 10^5^ and 10^7^ cells ml^−1^.

For endogenous tagging of *L. mexicana* genes, constructs and sgRNAs were generated using the PCR-based approaches previously described (Beneke et al., 2017; Dean et al., 2015), using the pLPOT (also called pLrPOT) (Edwards et al., 2018) series of plasmids as the PCR template. *L. mexicana* were transfected and subjected to drug selection as previously described (Dean et al., 2015). For endogenous tagging with HaloTag, a new pLPOT variant with HaloTag and blasticidin deaminase was generated.

The *L. mexicana* cell lines with deletion of both alleles of flagellum and FAZ proteins were generated using the PCR-based approach previously described, using the pT series of plasmids as the PCR template (Beneke et al., 2017). All deletion cell lines have been previously been characterised: ΔPFR2, ΔIC140 and ΔHydin in Beneke et al. (2019) and ΔFAZ5 in Sunter et al. (2019).

Microscopy was performed with an Axio Observer A1 (Zeiss) microscope with incubator chamber using a 63× NA 1.4 Ph3 objective or a 100× NA 1.4 without phase ring using a 120 V metal halide fluorescence light sources (Zeiss, HXP 120 V). Standard fluorescent microscopy was performed with an mRFP (Zeiss, 63HE) or GFP (ThorLabs, MDF-GFP2) filter cube.

To synchronously record red and green fluorescence at high frame rate, we used an OptoSplit II (Cairn Research) optical splitter. A custom microscope filter cube was fitted with a dual band (green and red) dichroic (Chroma Technology, 59022bs) and a dual band (blue and yellow) excitation filter (Chroma Technology, 59022x). The optical splitter was fitted with a filter cube with a 565 nm dichroic filter (Chroma Technology, T565lpxr) and green reflected and red transmitted light filters (Chroma Technology, ET520/40 m and ET632/60 m). When using white epi-illumination, this results in the splitter projecting a green and red fluorescence image onto two halves of a single camera, a Neo 5.5 (Andor). A 2019 mm focal length lens was used in the red light path for focus correction.

Synchronously recording red fluorescence and brightfield microscopy (in green) was possible because the samples do not emit green fluorescence. Therefore, when using white epi-illumination and trans-illumination through a green filter (ThorLabs, Astronomy Green), this set-up resulted in the splitter projecting a green brightfield and red fluorescence image onto the two halves of the camera.

The red and green images must be aligned to generate composite images. Images for calibration of position and scale/magnification were captured using multi-wavelength fluorescent beads (TetraSpeck 0.1 µm Microspheres, Invitrogen T7279); then the red and green images were aligned using the same approach we previously used for multi-focal plane microscopy (Walker and Wheeler, 2019).

For microscopy of cells adhered to glass, a sample of cells from late logarithmic growth (0.5×10^7^ to 1.0×10^7^ cells ml^−1^) were taken from culture, washed and placed on a slide then imaged live as previously described (Halliday et al., 2018).

For microscopy of free-swimming and naturally behaving cells, samples were taken from cultures in late logarithmic growth. To reduce cell adherence, glass slides were blocked with BSA prior to use by immersion in 1% BSA in distilled water for 30 s, followed by three washes in distilled water and air drying. 1 μl cells from culture in normal culture medium were applied to a 2 cm by 1 cm rectangle marked on a slide and coverslip using a hydrophobic pen, resulting in a liquid layer 2 μm thick. As the flagellum beat amplitude is typically >2 μm, this reduces the chance of the cell rotating such that the beat plane is perpendicular to the focal plane. This is, however, an artificial confinement and the confining surfaces introduce skin friction drag.

In order to generate the plots of tangent angle at different distances along the flagellum over time, flagella were automatically traced from videomicrographs using ImageJ. We used the intensity thresholding, skeletonisation and tracing scheme we previously developed for phase-contrast videos (Walker and Wheeler, 2019). The skeleton midline is on the pixel grid; this was then sampled at 3 pixels. The angle between the first and fifth points were taken as the cell orientation in each frame and graphs of tangent angle are shown with a 17-pixel rolling average. For tracing the fluorescence in high speed videos, the approach was adapted such that red fluorescent signal (either SMP1::mCh or mCh::RSP4/6) was subject to intensity thresholding following a correction for photobleaching. Flagella were digitally straightened using the ImageJ straighten tool using the flagellum midline from the red fluorescence as the line selection for straightening both the green and red fluorescence images.

For SEM cytoskeleton preparations, cells were harvested from culture by centrifugation (800 ***g*** for 3 min), washed three times in PBS, settled on coverslips in 24-well plates, washed with 0.1% NP-40 in PBS, washed three times in PBS and then fixed in 2.5% glutaraldehyde in PBS for 2 h at room temperature and then at 4°C overnight. Fixed cells were washed three times for 5 min in PBS and incubated in 1% osmium tetroxide in PBS at 4°C for 1 h in darkness. Samples were then washed three times with water for 5 min and ethanol dehydrated. Sample drying was completed by critical point drying using an Autosamdri-815 (Tousimis). Coverslips were then sputter gold coated for 60 s. Samples were imaged at around 20,000 to 35,000× on a JSM-6390 SEM (JEOL) with an Everhart-Thornley secondary electron detector using an EHT target of 2 kV and a 20 µm aperture.

## Supplementary Material

Supplementary information

Reviewer comments
