# Supplementary material for: The single flagellum of Leishmania has a fixed polarisation of its asymmetric beat
Source: J Cell Sci. 2020 Oct 22;133(20):jcs246637. doi: 10.1242/jcs.246637 (PMC7595685; doi:10.1242/jcs.246637)
Supplement: Supplementary information [file joces-133-246637-s1.pdf]

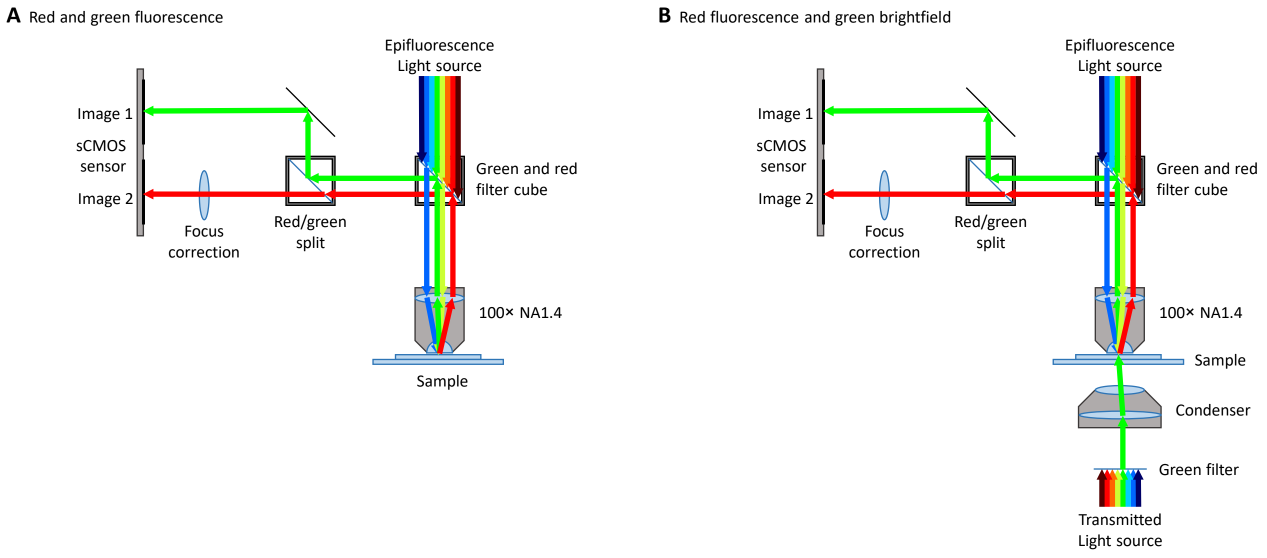

Figure S1. Summary of the microscope light paths for dual colour high resolution high frame rate microscopy. A. The light path configuration for simultaneous green and red fluorescence visualisation. **B.** The light path for red fluorescence visualised simultaneously with bright field transmitted light. The light path is the same, except that the sample does not emit green fluorescence and a transmitted light source through a green filter is used.

Journal of Cell Science • Supplementary information

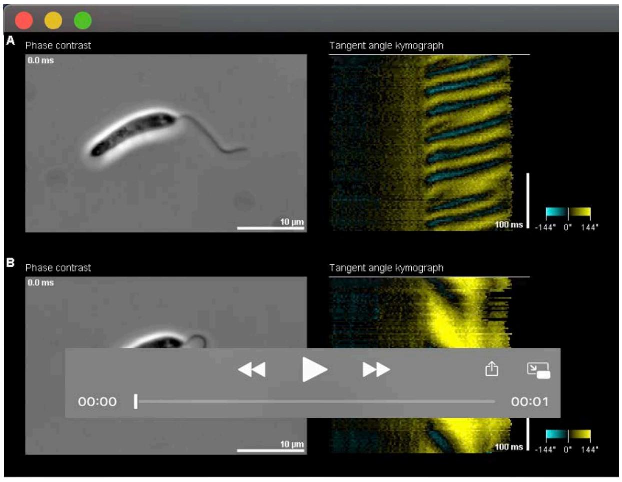

Movie 1. Animated version of Figure 2A-D.

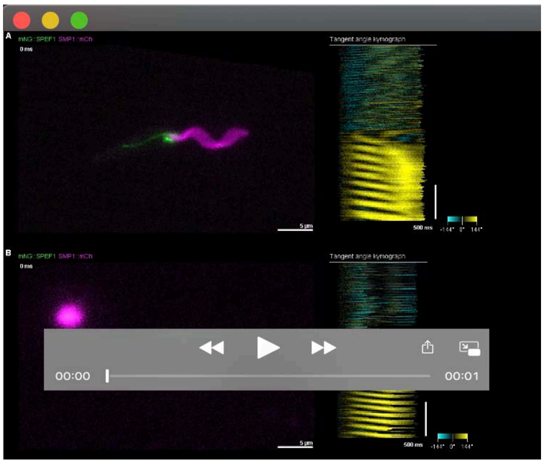

Movie 2. Animated version of Figure 3.

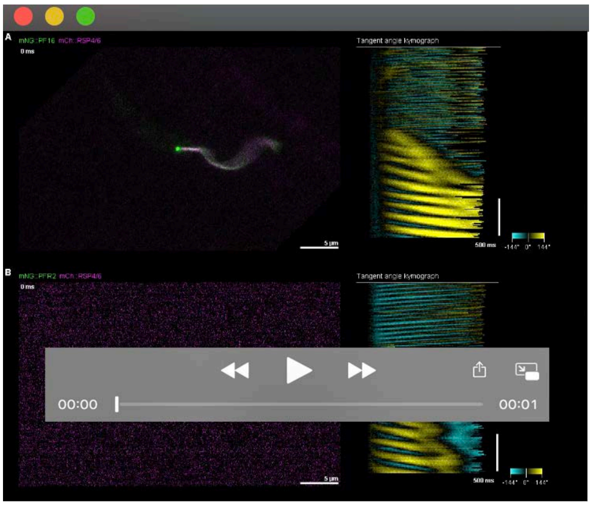

Movie 3. Animated version of Figure 4.

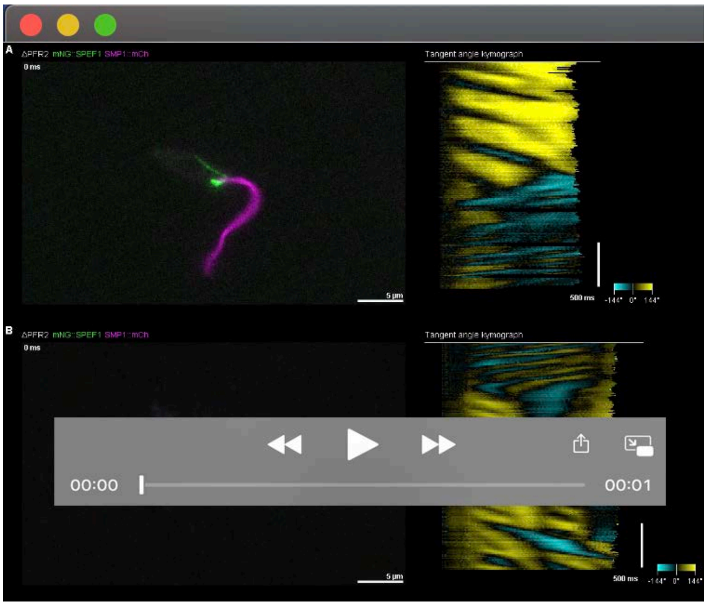

Movie 4. Animated version of Figure 7A-F.

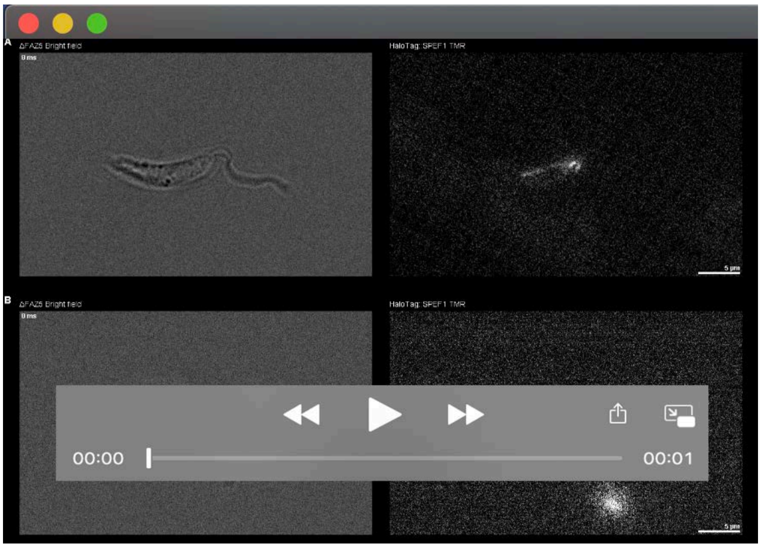

Movie 5. Animated version of Figure 7G-H.

Journal of Cell Science • Supplementary information
