# Supplementary material for: The single flagellum of Leishmania has a fixed polarisation of its asymmetric beat
Source: J Cell Sci. 2020 Oct 22;133(20):jcs246637. doi: 10.1242/jcs.246637 (PMC7595685; doi:10.1242/jcs.246637)
Supplement: Reviewer comments [file joces-133-246637_review_history.pdf]

The single flagellum of *Leishmania* has a fixed polarisation of its asymmetric beat

Ziyin Wang, Tom Beneke, Eva Gluenz and Richard John Wheeler
DOI: 10.1242/jcs.246637

Editor: David Stephens

Review timeline

Original submission:	21 March 2020
Editorial decision:	12 May 2020
First revision received:	21 July 2020
Editorial decision:	24 August 2020
Second revision received:	14 September 2020
Accepted:	17 September 2020

Original submission

First decision letter

MS ID#: JOCES/2020/246637

MS TITLE: The single flagellum of Leishmania has a fixed polarisation of its asymmetric beat

AUTHORS: Ziyin Wang, Tom Beneke, Eva Gluenz, and Richard Wheeler
ARTICLE TYPE: Research Article

We have now reached a decision on the above manuscript.

To see the reviewers' reports and a copy of this decision letter, please go to: https://submit-jcs.biologists.org and click on the 'Manuscripts with Decisions' queue in the Author Area. (Corresponding author only has access to reviews.)

As you will see, the reviewers raise a number of substantial criticisms that prevent me from accepting the paper at this stage. They suggest, however, that a revised version might prove acceptable, if you can address their concerns. If you think that you can deal satisfactorily with the criticisms on revision, I would be pleased to see a revised manuscript. We would then return it to the reviewers.

In particular, the point around the dynamics of the transitions (point 2 from reviewer 1) is quite important but we appreciate that you might not have been able to obtain the longer time frame data to support this. While anything you could do to address this point would be useful, I would not consider it essential should you not have the optimal longer timeframe data sets available. In terms of the other comments, I would hope that you can address these through clarifications to the text and figures and possibly some further analysis of existing data sets.

We are aware that you may currently be unable to access the lab to undertake experimental revisions. If it would be helpful, we encourage you to contact us to discuss your revision in greater detail. Please send us a point-by-point response indicating where you are able to address concerns raised (either experimentally or by changes to the text) and where you will not be able to do so within the normal timeframe of a revision. We will then provide further guidance. Please also note that we are happy to extend revision timeframes as necessary.

© 2020. Published by The Company of Biologists under the terms of the Creative Commons Attribution License (https://creativecommons.org/licenses/by/4.0/).

Please ensure that you clearly highlight all changes made in the revised manuscript. Please avoid using 'Tracked changes' in Word files as these are lost in PDF conversion.

I should be grateful if you would also provide a point-by-point response detailing how you have dealt with the points raised by the reviewers in the 'Response to Reviewers' box. Please attend to all of the reviewers' comments. If you do not agree with any of their criticisms or suggestions please explain clearly why this is so.

Reviewer 1

Advance summary and potential significance to field

Leishmania is one of the emerging model systems for studies of ciliary and flagellar motility. It is distinguished by its genetic tractability, and relevance to a number of known human diseases. Unusually, it is capable of both the so-called ‘ciliary’ beat, as well as the ‘flagellar beat’, and switches readily between the two modes.

Here, the authors used an elegant combination of novel live-cell high-speed imaging techniques, computer-aided analyses, and genetic tagging of fluorescent reporters to deduce that the source of symmetry-breaking of the asymmetric beat pattern is constrained to within the axoneme itself. I recommend this work enthusiastically for publication in JCS. I only have some minor comments and recommendations.

Comments for the author

1. The authors discuss the orientation of the central pair with respect to the beat plane - but in some other species of flagellates (e.g. Chlamydomonas) the central pair has been reported to rotate - does the CP rotate in this system?

2. Is it known what triggers transitions between the symmetric and asymmetric modes? Is there a propensity for selection of one over the other? Are the dynamics of these transitions consistent over multiple events? (if authors have a few examples they could check to see if some common timescale emerges - from the videos/figures shown it seems this switching happens quite quickly, what sets this timescale. Maybe worth summarising any relevant results from the Edwards et al paper here?)

3. The beat pattern itself is also a form of polarisation - any ideas why radii of curvature are so different between the symmetrical vs asymmetrical cases? Is there anything conclusive how the beat patterns are changed in the deletion mutants - esp the PFR2?

4. What sets the flagellar beat plane in Leishmania? is this known - it seems that in order organisms a number of structural asymmetries within the axoneme and asymmetries in the attachment between flagellum and cell body have been identified, but their functional roles are still unclear.

5. In relation to the comments in 319-321: What is the role of the PFR and the FAZ, if not to influence beat polarisation? A few more words about this may be helpful. Why are these structures present in these branches of eukaryotes? Given that the deletion mutants show apparently more ‘noisy’ beating dynamics could these additional structures stabilise the beat pattern in some way?

6. Relationship between beat polarisation and free-swimming dynamics - the authors argue in the discussions (293-294) that this could possibly constrain the tactic mechanisms of the cell? can the authors elaborate on this point? It is still possible to turn in all directions with a chiral beat pattern - the fluorescence data show that not all parts are in focus at the same time - so I’m not sure where the limitation in movement direction would come from?

minor comments
a clearer definition of ‘polarisation’, particularly ‘polarisation of an asymmetric beat’ in this context, perhaps with labels referencing the body plan of the cell - at least a few more words around lines 38-39 - this is just in case it is not clear to a general readership.

© 2020. Published by The Company of Biologists under the terms of the Creative Commons Attribution License (https://creativecommons.org/licenses/by/4.0/).

lines 78-79 - reference to ‘invert the symmetry of the beat’, do the authors perhaps mean ‘invert the polarisation of the beat’?

the fluorescence imaging is very nice - but are there no distinguishing extracellular features on the Leishmania body at all? Could something as simple as cell shape - which looks like it generally has some curvature, not be an indicator of polarity?

lines 233-251: which type of beat is more common in the deletion mutant? tip-to-base or base-to-tip? the writing seems to be inconsistent in this paragraph? Please check carefully.

figure 5 - consider relabelling, don’t refer to directions A,B when there are already two identical labels A,B for the figures… Likewise in figure 6?

Reviewer 2

Advance summary and potential significance to field

This manuscript studies the asymmetric motion of the single flagellum of Leishmania. Leishmania can switch from a symmetric to asymmetric flagellar movement: high-frequency symmetric tip-to-base wavefronts are transformed into asymmetric low-frequency base-to-tip wavefronts. By using high frame rate dual colour fluorescence microscopy to visualise intracellular and intraflagellar structures together with waveform analysis, this study showed that the asymmetric beat of Leishmania has a “fixed polarisation”, i.e. the asymmetry of the beat is conserved in relation to the cell body orientation (occurring always at same side of the cell body). Most importantly, the authors investigate whether extra-axonemal asymmetric structures play an important role in shaping the asymmetric beat. For this, they looked at mutants lacking a major component of the paraflagellar rod and the asymmetrically-positioned flagellum attachment zone. It is concluded that these asymmetric structures are not required for the asymmetric beat. Mutants lacking the inner dynein arm and Hydin (required to prevent central pair rotation in the axoneme) are also studied. These mutants are non-motile and have a preferred bend direction towards one side of the cell (opposite to the asymmetric beating side) with the paraflagellar rod on the outside of the spiral coil – this was further confirmed with scanning electron microscopy. Finally, an attempt is made to connect these results with the static and dynamic curvature of Chlamydomonas (Geyer 2016). The manuscript is well written and structured with clear figures, videos and discussion of results. This study clearly contributes to important aspects flagellar asymmetries in Leishmania. However few important points should be addressed first.

Comments for the author

1) The authors build on the work of Chlamydomonas flagella (Geyer 2016). Greyer et al showed that the mathematical separation between static and dynamic curvature are indeed manifested independently within the flagellum by investigating distinct mutants. This is not the case here however as none of the mutants manifested the separation between the static and dynamic modes in the manuscript. This need clarifying. The static curvature is measured by time averaging the waveform. Subtracting this temporal bias from the original signal gives, by definition, the symmetric part of the signal, termed dynamical curvature (as in (Geyer 2016)). Thus the symmetric dynamical part arises via mathematical construction for any waveform, symmetric or not. Likewise, the definitions of static and dynamic curvatures could be made clearer as their biophysical independence is not an immediate consequence.

2) It is not clear what is gained from the (ΔIC140) or the central pair (ΔHydin) mutants – in contrast with the other mutants which have a clear motivation from their asymmetric contribution to flagellum structures. Why did the authors look at (ΔIC140) and (ΔHydin) mutants given that the coiled shape was previously reported? Even if the coil direction was in the same side as the asymmetric beat, it is not clear how this information could inform on the ability of the cell to produce asymmetric wavefronts, given the vast number of other possibilities (lack of other components) that could provide similar effect.

© 2020. Published by The Company of Biologists under the terms of the Creative Commons Attribution License (https://creativecommons.org/licenses/by/4.0/).

3) The introduction describing the differences between ciliary and flagellar beating is not supported by key texts/references and it debatable. There are a number of instances in which strong claims are not supported by references: line 50 - “Structural asymmetries for keeping bending in a plane are well-characterised.”; line 288 – “Most organisms appear to have a fixed polarisation of the asymmetric beat,”, among others.

4) The paper attempts to place the work in the wider context of asymmetric beating flagella however in addition to Leshmanina, only Chlamydomonas is mentioned. There are a number of Eukaryotic flagella that beat asymmetrically, but is not considered a cilium. Could the authors comment on the relevance of this study to the wider context? Asymmetric beating flagella are critical for chemotaxis for example.

5) The terms “curvature” and “tangent angle” are used interchangeably but should be avoided. The curvature of the waveform was not measured for any cell and its interchangeable use is misleading, including figure labels, captions and conclusions. All figures refer to curvature plots but the tangent angle is depicted instead. This leads to inconsistent statements: line 133 “the static curvature is near-linear”. Linear tangent angle implies instead a constant curvature.
These definitions need revision, as well as more details on how they were obtained in Methods, including any associated errors to these measurements.

6) More details in Materials and Methods on how the beating frequency was evaluated, the tangent angle, discretization used for the flagellar points, errors etc. Are these observations consistent with previous reports by (Gadelha 2007) on the wavefront characteristics? Will this data be made available? Which cells are free-swimming and tethered in the figures? Also a 2um liquid layer depth is very small for the flagellum to beat. Please comments on how this may interfere with the results obtained.

7) Fig 3 shows some blurred regions in the image. Is this because of the low frame rate 100Hz or the out of plane motion? How does this influence your measurements given it introduces biases in the measured wavefront, potentially influencing the tangent angle. For cells beating at 25Hz, for example, 100Hz frame rate can only provide 4 time-points within the period.

8) It is argued that “It is unknown whether a single Leishmania flabellum has a fixed polarisation for its asymmetric beat” and that “Leishmania cell is near-axially symmetric meaning cell orientation is not visible”. However for many of the cells shown in the manuscript the body had an asymmetric shape that could be tracked. This is obviously not true for all cases, as shown in (Gadelha 2007). It is worth noting in the manuscript that large body shape variability means that body shape cannot be used as a reliable proxy of asymmetry between different cells motivating this important study.

9) There are few typos in the text and captions. Fig 3B blue label is not showing the membrane in the figure etc.

First revision

<u>Author response to reviewers' comments</u>

We would like to thank the editor and both reviewers for their support and their detailed constructive criticism. We have made many changes to the text (with key passages altered highlighted in yellow) and added two new analyses to address the various comments.

Reviewer 1
Advance summary and potential significance to field...
“Leishmania is one of the emerging model systems for studies of ciliary and flagellar motility. It is distinguished by its genetic tractability, and relevance to a number of known human diseases. Unusually, it is capable of both the so-called ‘ciliary’ beat, as well as the ‘flagellar beat’, and switches readily between the two modes.

© 2020. Published by The Company of Biologists under the terms of the Creative Commons Attribution License (https://creativecommons.org/licenses/by/4.0/).

Here, the authors used an elegant combination of novel live-cell high-speed imaging techniques, computer-aided analyses, and genetic tagging of fluorescent reporters to deduce that the source of symmetry-breaking of the asymmetric beat pattern is constrained to within the axoneme itself. I recommend this work enthusiastically for publication in JCS. I only have some minor comments and recommendations."

Reviewer 1 Comments for the author...
"1. The authors discuss the orientation of the central pair with respect to the beat plane - but in some other species of flagellates (e.g. Chlamydomonas) the central pair has been reported to rotate - does the CP rotate in this system?"
The central pair orientation in trypanosomatids is fixed relative to the outer doublets. This has previously been characterised in detail for Trypanosoma brucei (Gadelha et al., 2006), and this study also analysed two species closely related to Leishmania mexicana: Leishmania major and Crithidia fasciculata. This was indicated in the results (line 137) but has been added to the introduction for clarity too.
Thank you for highlighting this result - this paper also showed (in T. brucei) that the central pair remains in a fixed orientation following PFR2 knockdown by RNAi, which further informs the PFR2 deletion result here.
"2. Is it known what triggers transitions between the symmetric and asymmetric modes? Is there a propensity for selection of one over the other? Are the dynamics of these transitions consistent over multiple events? (if authors have a few examples they could check to see if some common timescale emerges - from the videos/figures shown it seems this switching happens quite quickly, what sets this timescale. Maybe worth summarising any relevant results from the Edwards et al paper here?)"
We have not yet been able to identify any clear trigger for symmetric/asymmetric switching – it seems random in normal culture. We do see a bias towards switching from symmetric to asymmetric in our dataset. This may be an effect of microscopy illumination, or may simply be a selection bias (it is typically easier to see that cells which are undergoing the symmetrical beat are not stuck to the slide).
The high speed videos are unfortunately too short in length to be able to analyse the interval between switches. From lower frame rate videos we estimate that switches tend to occur around every 10 s to 1 min, but these videos do not have the necessary frame rate to classify the flagellum beat type with any confidence. We do hope to upgrade our camera technology in the future to address these questions though.
In terms of the dynamics of the switch itself, we revisited the dataset which has ~20 such switches. It has some variability, it typically takes one or two, but sometimes up to five, base-to-tip beats for the tip-to-base beats to fully stop. We suspect that there may be dependency on the flagellum length, which is very variable in Leishmania populations (see Wheeler, Gluenz and Gull 2011), and as such would need a very large sample size to draw concrete conclusions – this is unfortunately not feasible at this time, so we have added a weaker qualitative statement to the text.
"3. The beat pattern itself is also a form of polarisation - any ideas why radii of curvature are so different between the symmetrical vs asymmetrical cases? Is there anything conclusive how the beat patterns are changed in the deletion mutants - esp the PFR2?"
This is certainly interesting – I get the impression that the reviewer is interested in whether the PFR could be defining bend radius, and whether bend radius is altered in the PFR2 deletion.
We revisited the dataset and measured the tightest curvature over the beat cycle for the power and recovery strokes in 'wild-type' (only tagged), PFR2 deletion and FAZ5 deletion mutants. This showed no change in curvature in these mutants, which points to the asymmetry in curvature originating from the axoneme and not the axoneme associated structures. This new analysis has been added to Figure 7.
"4. What sets the flagellar beat plane in Leishmania? is this known - it seems that in order organisms a number of structural asymmetries within the axoneme and asymmetries in the attachment between flagellum and cell body have been identified, but their functional roles are still unclear."
Again, this is a very interesting question. To our knowledge there is no evidence for which Leishmania axonemal or accessory structures are required for planar vs. three dimensional flagellar movement – none of the mutants here gave a particular appearance of three-dimensionality leaving this unclear.

© 2020. Published by The Company of Biologists under the terms of the Creative Commons Attribution License (https://creativecommons.org/licenses/by/4.0/).

However, it is valuable to note that we did not see the Hydin, IC140, PFR or FAZ deletion mutants deviating from the expected plane of bending. We have added comments about this to various points in the text. This links to Reviewer 2 point 3.
"5. In relation to the comments in 319-321: What is the role of the PFR and the FAZ, if not to influence beat polarisation? A few more words about this may be helpful. Why are these structures present in these branches of eukaryotes? Given that the deletion mutants show apparently more 'noisy' beating dynamics, could these additional structures stabilise the beat pattern in some way?"
We would suggest the FAZ most likely is involved as a mechanical anchoring type structure, though have no direct evidence for this here – recent work by Jack Sunter comes closer to addressing this question and I would like to avoid over-interpretation here.
For the PFR this is complex. Naively, a simple mechanical/structural role might be assumed based on its appearance, however our results here suggest that is unlikely to be the case (at least from the point of view of origins of asymmetry). The PFR is a complex structure with PFR2 being a major structural component - this means the PFR2 deletion phenotype is the combined effect of the loss of >100 proteins from the flagellum greatly complicating any interpretation.
We have added a short note to the discussion emphasising that both of these structures are important for normal beating, but not for normal polarisation.
"6. Relationship between beat polarisation and free-swimming dynamics - the authors argue in the discussions (293-294) that this could possibly constrain the tactic mechanisms of the cell? can the authors elaborate on this point? It is still possible to turn in all directions with a chiral beat pattern - the fluorescence data show that not all parts are in focus at the same time - so I'm not sure where the limitation in movement direction would come from?"
We agree that this needs some clarification. This is related to a point from Reviewer 2 which we think is best addressed by comparison with smaller asymmetries in beating involved in sperm chemotaxis. The argument we were pushing does not preclude chemotaxis mechanisms through more subtle modulations of beat asymmetry/chirality in the symmetrical tip-to-base beat, it also does not mean that the asymmetrical base-to-tip beat cannot achieve any target rotation (though may have to rotate >180 degrees to do so). It speaks only to the immediate response a cell may make to a chemotactic signal, interaction with a surface, etc. – it can only turn in one direction. This section of the discussion has been rewritten to better consider these points.
Minor comments
"a clearer definition of 'polarisation', particularly 'polarisation of an asymmetric beat' in this context, perhaps with labels referencing the body plan of the cell - at least a few more words around lines 38-39 - this is just in case it is not clear to a general readership."
We have added earlier references to Figure 1A and B when we introduce the symmetric and asymmetric beat types and specifically highlighted how a reverse polarisation in 1B would give reversed fluid flow and cell rotation – this should help a general reader.
"lines 78-79 - reference to 'invert the symmetry of the beat', do the authors perhaps mean 'invert the polarisation of the beat'?"
We agree that this suggestion is clearer. We tried to keep the terminology consistent with careful use of polarisation and symmetry/asymmetry, but this one slipped through.
"the fluorescence imaging is very nice - but are there no distinguishing extracellular features on the Leishmania body at all? Could something as simple as cell shape - which looks like it generally has some curvature, not be an indicator of polarity?"
Leishmania do indeed tend to have a small curvature, although it tends not to be visible in shorter cells (G1 cells). In addition, there is no data which links the orientation of cell curvature to the orientation of the flagellar structures – the key advantage of our approach here is that we can map the central pair orientation, PFR position, axoneme orientation etc. via SPEF1 and PFR2 fluorescence. This is related to a point by Reviewer 2 and we have added a clearer explanation to the introduction.
"lines 233-251: which type of beat is more common in the deletion mutant? tip-to-base or base-to-tip? the writing seems to be inconsistent in this paragraph? Please check carefully."
Thank you for spotting this error – we had inadvertently swapped tip-to-base and base-to-tip in one sentence.
"figure 5 - consider relabelling, don't refer to directions A,B when there are already two identical labels A,B for the figures… Likewise in figure 6?"
We agree that this was a poor choice of labels. We have updated this to use polarisations 1 and 2 rather than directions A and B. This should remove any confusion referring to panels and also make the use of the term 'polarisation' completely consistent (see first minor comment).

© 2020. Published by The Company of Biologists under the terms of the Creative Commons Attribution License (https://creativecommons.org/licenses/by/4.0/).

Reviewer 2
Advance summary and potential significance to field...
"This manuscript studies the asymmetric motion of the single flagellum of Leishmania. Leishmania can switch from a symmetric to asymmetric flagellar movement: high-frequency symmetric tip-to-base wavefronts are transformed into asymmetric low-frequency base-to-tip wavefronts. By using high frame rate dual colour fluorescence microscopy to visualise intracellular and intraflagellar structures together with waveform analysis, this study showed that the asymmetric beat of Leishmania has a "fixed polarisation", i.e. the asymmetry of the beat is conserved in relation to the cell body orientation (occurring always at same side of the cell body). Most importantly, the authors investigate whether extra-axonemal asymmetric structures play an important role in shaping the asymmetric beat. For this, they looked at mutants lacking a major component of the paraflagellar rod and the asymmetrically-positioned flagellum attachment zone. It is concluded that these asymmetric structures are not required for the asymmetric beat. Mutants lacking the inner dynein arm and Hydin (required to prevent central pair rotation in the axoneme) are also studied. These mutants are non-motile and have a preferred bend direction towards one side of the cell (opposite to the asymmetric beating side) with the paraflagellar rod on the outside of the spiral coil – this was further confirmed with scanning electron microscopy. Finally, an attempt is made to connect these results with the static and dynamic curvature of Chlamydomonas (Geyer 2016). The manuscript is well written and structured with clear figures, videos and discussion of results. This study clearly contributes to important aspects flagellar asymmetries in Leishmania. However a few important points should be addressed first."

Reviewer 2 Comments for the author...
"1) The authors build on the work of Chlamydomonas flagella (Geyer 2016). Greyer et al showed that the mathematical separation between static and dynamic curvature are indeed manifested independently within the flagellum by investigating distinct mutants. This is not the case here however as none of the mutants manifested the separation between the static and dynamic modes in the manuscript. This need clarifying. The static curvature is measured by time averaging the waveform. Subtracting this temporal bias from the original signal gives, by definition, the symmetric part of the signal, termed dynamical curvature (as in (Geyer 2016)). Thus the symmetric dynamical part arises via mathematical construction for any waveform, symmetric or not. Likewise, the definitions of static and dynamic curvatures could be made clearer as their biophysical independence is not an immediate consequence."
Using the terms static and dynamic curvature is very convenient for describing our results, and we do feel similar biology is likely to underlie the Leishmania swimming behaviour. Having said that, we do see that usage of the terms presented a mathematical construction as a biological result – this was unclear.
We have rephrased the results to make it clear that our terminology derives from a mathematical separation of static/dynamic curvature. However, as we think the evidence from Chlamydomonas is informative for interpreting our results we have retained carefully rephrased conclusion/discussion points.
"2) It is not clear what is gained from the (ΔIC140) or the central pair (ΔHydin) mutants – in contrast with the other mutants which have a clear motivation from their asymmetric contribution to flagellum structures. Why did the authors look at (ΔIC140) and (ΔHydin) mutants given that the coiled shape was previously reported? Even if the coil direction was in the same side as the asymmetric beat, it is not clear how this information could inform on the ability of the cell to produce asymmetric wavefronts, given the vast number of other possibilities (lack of other components) that could provide similar effect."
These mutants were selected as they are the only mutants known in Leishmania which have lost the ability to beat the flagellum but the majority of the population still have pronounced curvature of the flagellum – therefore making it possible to observe asymmetries in bending in the absence of normal beating. We also note that deletion of the Leishmania MBO2 ortholog does not phenocopy the Chlamydomonas mbo2 mutant.
Our previous work has involved 100 deletion mutants so we are confident that there are not a vast number of other possibilities. Furthermore, ΔHydin mutant has a disrupted central pair, therefore has a defect in one of the canonical axoneme asymmetries.
We fully agree that these mutants are not informative for how a cell can generate asymmetric wavefronts vs. symmetrical wavefronts – the flagella can't produce waves! The value is a little more subtle – asking whether, for these flagella which bend but cannot produce waves, is the polarity constant. We have clarified this subtlety in the text.

© 2020. Published by The Company of Biologists under the terms of the Creative Commons Attribution License (https://creativecommons.org/licenses/by/4.0/).

"3) The introduction describing the differences between ciliary and flagellar beating is not supported by key texts/references and it debatable. There are a number of instances in which strong claims are not supported by references: line 50 - "Structural asymmetries for keeping bending in a plane are well-characterised."; line 288 – "Most organisms appear to have a fixed polarisation of the asymmetric beat,", among others."
Regarding line 50: The aim of this introductory point was to emphasise why symmetry breaking is necessary for observed flagellar motions, however in focusing on that point we over-simplified how it presented existing data. This has now been rephrased to better reflect the level of experimental evidence.
Regarding line 288: We have lengthened this discussion to include more evidence to support our point. We note that terminology in this field is complicated by the use of language – the phrase 'flagellar/ciliary reversal' is often used, often without evidence for actual waveform shape. Where there is evidence for waveforms, such as Chlamydomonas, 'flagellar reversal' involves neither reversal of polarity nor reversal of propagation of waves along the flagellum.
"4) The paper attempts to place the work in the wider context of asymmetric beating flagella, however in addition to Leshmanina, only Chlamydomonas is mentioned. There are a number of Eukaryotic flagella that beat asymmetrically, but is not considered a cilium. Could the authors comment on the relevance of this study to the wider context? Asymmetric beating flagella are critical for chemotaxis for example."
I assume that the reviewer is referring to asymmetries in sperm flagella as a prominent example of smaller asymmetries required for chemotaxis? It would have been useful to specifically indicate key species/cells or studies as this comment is unclear.
As we are focusing on a large asymmetry (ie. with greater similarity to the ciliary beat) we have focused on comparison to strongly asymmetric ciliary beats for the introduction. However, the smaller asymmetries which are used to modulate sperm swimming may be related – it is certainly notable that sperm trajectories tend to always curve in one direction and that helical klinotaxis is based on the resulting helical paths. We have added a short consideration of this point to the discussion.
"5) The terms "curvature" and "tangent angle" are used interchangeably but should be avoided. The curvature of the waveform was not measured for any cell and its interchangeable use is misleading, including figure labels, captions and conclusions. All figures refer to curvature plots but the tangent angle is depicted instead. This leads to inconsistent statements: line 133 "the static curvature is near-linear". Linear tangent angle implies instead a constant curvature. These definitions need revision, as well as more details on how they were obtained in Methods, including any associated errors to these measurements."
Thank you for this comment, this is certainly an important necessary clarification – we have carefully corrected use of curvature vs. tangent angle, especially in the light of adding flagellar curvature measurements for the various mutants (see comment from Reviewer 1).
As also mentioned below, we have added more detail to the methods about precisely how tangent angles were calculated and presented. We have also added analysis from many more cells to show the population variation (Figure 2G, H).
"6) More details in Materials and Methods on how the beating frequency was evaluated, the tangent angle, discretization used for the flagellar points, errors etc. Are these observations consistent with previous reports by (Gadelha 2007) on the wavefront characteristics? Will this data be made available? Which cells are free-swimming and tethered in the figures? Also a 2um liquid layer depth is very small for the flagellum to beat. Please comments on how this may interfere with the results obtained."
Beat frequency was only approximately determined, by simple counts of number of cycles in number of frames. The precise method for tracing the flagellum to determine tangent angle etc. was exactly as we previously described (Walker and Wheeler, 2019) – however we have added the key discretisation parameters to the method for clarity.
Our observations are in a different species (L. mexicana) to Gadelha et al. 2007 (which used L. major as an example Leishmania species), however the cell morphology, beat frequency, flagellum curvature etc. are comparable. Note that we have not made claims from precise measurement of beat parameters here (see also comment 7) – the low framerate for the fluorescence videos limits the quality of analysis that would be possible, particularly for the tip-to-base beat.
It is unclear which data that the reviewer would like to be made available. The video data is significantly larger than the maximum size that can be submitted to general purpose repositories like Dryad or Figshare – however this data would be made available in the spirit of the JCS journal policies.

© 2020. Published by The Company of Biologists under the terms of the Creative Commons Attribution License (https://creativecommons.org/licenses/by/4.0/).

We have also added some discussion of the limitations arising from using a thin fluid layer to the methods section.
“7) Fig 3 shows some blurred regions in the image. Is this because of the low frame rate 100Hz or the out of plane motion? How does this influence your measurements given it introduces biases in the measured wavefront, potentially influencing the tangent angle. For cells beating at 25Hz, for example, 100Hz frame rate can only provide 4 time-points within the period.”
This is indeed because of the 100Hz frame rate vs. the ~25Hz tip-to-base flagellar-type beat. As the aim of this work was to determine orientation of beat asymmetry using fluorescence we had to balance exposure time for fluorescence signal to noise with framerate to visualise the beat. We did not draw any conclusions from precise tangent angle as we agree that this comparatively low framerate could introduce biases in the wavefront of the higher frequency tip-to-base symmetrical beat. However, this does not affect any of our conclusions: This visualisation was primarily used as a convenient way to summarise flagellum movement in a static figure, analysis of the orientation of asymmetric beats was made from the video data.
“8) It is argued that “It is unknown whether a single Leishmania flabellum has a fixed polarisation for its asymmetric beat” and that “Leishmania cell is near-axially symmetric meaning cell orientation is not visible”. However for many of the cells shown in the manuscript the body had an asymmetric shape that could be tracked. This is obviously not true for all cases, as shown in (Gadelha 2007). It is worth noting in the manuscript that large body shape variability means that body shape cannot be used as a reliable proxy of asymmetry between different cells, motivating this important study.”
This is similar to a comment by Reviewer 1 – Leishmania tend to have a curved cell shape in some cell cycle stages but as the reviewer notes it is not seen in all cells. It is also not known if the curvature has a consistent orientation and, if it does have a constant orientation, what that orientation is relative to the flagellum ultrastructure – which is of particular importance for this study. The text has been updated to include these points.
“9) There are few typos in the text and captions. Fig 3B blue label is not showing the membrane in the figure etc.”
We have corrected the error in Fig 3B the reviewer noted in addition to some other minor errors.

Second decision letter

MS ID#: JOCES/2020/246637

MS TITLE: The single flagellum of Leishmania has a fixed polarisation of its asymmetric beat

AUTHORS: Ziyin Wang, Tom Beneke, Eva Gluenz, and Richard Wheeler
ARTICLE TYPE: Research Article

We have now reached a decision on the above manuscript. One reviewer has raised some further points that I simply wanted you to have the opportunity to respond to and to amend the text if you felt it appropriate. There are no further experiments required nor adjustments to figures. They raise some interesting points and you might wish to consider minor revision to the discussion as a result. Subject to any amendments or a short note detailing why you do not consider this necessary, I would then be happy to accept your paper for publication.

To see the reviewers' reports and a copy of this decision letter, please go to: https://submit-jcs.biologists.org and click on the 'Manuscripts with Decisions' queue in the Author Area. (Corresponding author only has access to reviews.)

We are aware that you may currently be unable to access the lab to undertake experimental revisions. If it would be helpful, we encourage you to contact us to discuss your revision in greater detail. Please send us a point-by-point response indicating where you are able to address concerns raised (either experimentally or by changes to the text) and where you will not be able to do so within the normal timeframe of a revision. We will then provide further guidance. Please also note that we are happy to extend revision timeframes as necessary.

© 2020. Published by The Company of Biologists under the terms of the Creative Commons Attribution License (https://creativecommons.org/licenses/by/4.0/).

Please ensure that you clearly highlight all changes made in the revised manuscript. Please avoid using 'Tracked changes' in Word files as these are lost in PDF conversion.

I should be grateful if you would also provide a point-by-point response detailing how you have dealt with the points raised by the reviewers in the 'Response to Reviewers' box. Please attend to all of the reviewers' comments. If you do not agree with any of their criticisms or suggestions please explain clearly why this is so.

Reviewer 1

Advance summary and potential significance to field

See significance statement from previous report.

Comments for the author

i am broadly satisfied the authors have addressed most of the concerns raised in the previous round of reviews. For completeness, they may wish to consider the following

1. a recent preprint https://www.biorxiv.org/content/10.1101/2020.03.15.991331v1 considers a possible role of the PFR in defining the 3D beat pattern of euglena - perhaps a comparison between the two systems may be instructive.
2. the second comment is more critical - it seems to me that all cilia/eukaryotic flagella have a fixed polarisation - are the authors even aware of any system in which the polarisation is not fixed? (not sure if the paramecium example is true) To me, the result presented in this paper is therefore unsurprising - though of course it is still important to demonstrate this is true through rigorous experiments. However my point is that it has become increasingly apparent that even in cases where the beat pattern might be naively assumed to be symmetric, there exists some form of fixed direction for the static curvature component, mostly likely arising from heterogeneities in the axoneme. What does this work mean for understanding how the beat pattern is shaped? What do the PFR and other structures contribute to this control? Or rather the inverse,
beyond merely stating that the asymmetric waveform is polarised, emphasize which structures do not contribute to polarisation? Some overarching comments on these generic features of eukaryotic flagella may be helpful to elevate the paper.
3. I'm still unconvinced about the discussion on turning (324-332) - I'm not sure how the fixed polarization would restrict directed movement, whether near boundaries or during osmotaxis. Is there a concrete model that the authors can reference - even if in a different organism? The Leslie et al paper does not show any dynamic data and is therefore unconvincing as an argument for mechanism. From sperm to algae to bacteria microorganisms have many different ways of removing themselves from boundaries, whether by active reorientation, by buckling instabilities, or some combination of the above. The fact that the beat pattern has a fixed polarization should not really constrain the range of movement - but perhaps only change stereotypical reorientation dynamics and timescales. The authors are encouraged to compare their case with sperm rheotaxis - about which much more is known.

Some minor comments
1. line 131 - 'confirmation of the flagellum', presumably 'conformation' is meant.
2. lines 186-189 might need a rewrite as wording unclear

Reviewer 2

Advance summary and potential significance to field

The revised version of the manuscript incorporate many new and important aspects discussions and corrections. I believe this article reports and discuss important observations on asymmetric flagellar wave generation and I am confident that the community will appreciate this tremendously. Congratulations to all authors for this beautiful study.

© 2020. Published by The Company of Biologists under the terms of the Creative Commons Attribution License (https://creativecommons.org/licenses/by/4.0/).

Comments for the author

N/A

Second revision

Author response to reviewers' comments

Reviewer 1:
i am broadly satisfied the authors have addressed most of the concerns raised in the previous round of reviews. For completeness, they may wish to consider the following
>I would like to thank Reviewer 1 once again for their detailed comments. In line with the editor's suggestions we have made a few changes to the discussion of the results and the discussion section to address these further points.
1. a recent preprint https://www.biorxiv.org/content/10.1101/2020.03.15.991331v1 considers a possible role of the PFR in defining the 3D beat pattern of euglena - perhaps a comparison between the two systems may be instructive.
>The PFR has also previously been implicated in giving rise to three-dimensionality of flagellum movement in T. brucei, assuming a different preferred bending direction of the PFR and axoneme inferred from PFR structure (Hughes et al., 2012). The new Euglena work is based on a similar assumption (assuming the PFR is offset from the bending plane of the axoneme). However, neither of these provide direct evidence, like analysis of a PFR2 deletion mutant, for this PFR function.
>The phenomenon described for T. brucei and Euglena can't be occurring in Leishmania: They have a PFR and a near-planar beat (Gadelha et al., 2007; Walker and Wheeler, 2019). Interestingly, a closely-related species naturally lacking a large PFR (Crithidia deanei) (Gadelha et al., 2005) has a flagellar beat very similar to Leishmania (Gadelha et al., 2007) and our data here do not show a distinct gain in three-dimensionality on deletion of PFR2.
>While it cannot explain the complex three-dimensional movement of Euglena flagella, lateral attachment of the flagellum to the cell body along much of its length by the FAZ is also implicated in generating three-dimensionality of flagellum movement in T. brucei (Sun et al., 2018) . Consistent with this, my previous work also showed a T. brucei FAZ mutant which has a greater length of free flagellum (Hayes et al., 2014) has near-planar flagellar movement (Wheeler, 2017).
>This situation is further complicated by the complexity of the PFR structure, which houses over 150 proteins (Dean et al., 2017; Portman et al., 2009), so loss of the PFR isn't simply a biomechanical change.
>All-in-all we agree that understanding the PFR function is important and that the Euglena data is valuable. However, there is good evidence that this biomechanical function can't be occurring in Leishmania – I think that discussing this point (as above) takes up too much space for something which is arguably tangentially relevant.
2. the second comment is more critical - it seems to me that all cilia/eukaryotic flagella have a fixed
polarisation - are the authors even aware of any system in which the polarisation is not fixed? (not sure if the paramecium example is true) To me, the result presented in this paper is therefore unsurprising - though of course it is still important to demonstrate this is true through rigorous experiments. However my point is that it has become increasingly apparent that even in cases where the beat pattern might be naively assumed to be symmetric, there exists some form of fixed direction for the static curvature component, mostly likely arising from heterogeneities in the axoneme. What does this work mean for understanding how the beat pattern is shaped? What do the PFR and other structures contribute to this control? Or rather the inverse, beyond merely stating that the asymmetric waveform is polarised, emphasize which structures do not contribute to polarisation? Some overarching comments on these generic features of eukaryotic flagella may be helpful to elevate the paper.
>We agree that there is not strong evidence for a system where the polarisation of ciliary/strongly asymmetric beats are not fixed – our null hypothesis was that the Leishmania beat would be fixed. However, there seem not to be paired ultrastructural and beat orientation data to be confident.

© 2020. Published by The Company of Biologists under the terms of the Creative Commons Attribution License (https://creativecommons.org/licenses/by/4.0/).

We did not mean to place too much weight on Paramecium, that example is the result of deeper examination of the literature in response to previous comments. We have made some minor changes to more clearly state that the current view is that polarisation is likely fixed, although more through lack of evidence than direct evidence, and place our results and discussion more clearly in that context.
>We agree that we haven't fully explored the second point, that superficially symmetric waveforms may have a fixed direction of the static curvature. However our data (Figure 1G) shows that static curvature in the symmetric waveform, if present, is small – at most around 0.1 rad or 6 degrees at the flagellar tip. With the methods here, particularly with restrictions in accuracy of reconstruction of the high frequency tip-to-base beat given frame rate and fluorescence intensity limitations, we are not confident we can accurately measure an effect reliably.
>Finally, we have revisited the conclusions to emphasise the points you highlighted. We did previously draw conclusions about the broader relevance of FAZ (analogous to rootlets) and the PFR (as an extra-axonemal structure), but we've now emphasised that these features do not contribute to polarisation and made some comments on the universality of these types of features in flagella.
3. I'm still unconvinced about the discussion on turning (324-332) - I'm not sure how the fixed polarization would restrict directed movement, whether near boundaries or during osmotaxis. Is there a concrete model that the authors can reference - even if in a different organism? The Leslie et al paper does not show any dynamic data and is therefore unconvincing as an argument for mechanism. From sperm to algae to bacteria, microorganisms have many different ways of removing themselves from boundaries, whether by active reorientation, by buckling instabilities, or some combination of the above. The fact that the beat pattern has a fixed polarization should not really constrain the range of movement - but perhaps only change stereotypical reorientation dynamics and timescales. The authors are encouraged to compare their case with sperm rheotaxis - about which much more is known.
>I feel this is a situation where text we intend as an easy to understand, but hypothetical, example is being interpreted as a rigorous result. Our intent is to highlight that this limitation in flexibility of flagellum beat type might have functional consequences – however, as pointed out, there is a wide range of more complex dynamics which are likely to also contribute. The theoretical/biophysical background for Leishmania is too limited to draw any strong conclusions, I am also hesitant to use sperm rheotaxis examples because of the complications of considering a puller rather than a pusher swimmer. Again, we've altered the discussion to clarify these points.
Some minor comments
1. line 131 - 'confirmation of the flagellum', presumably 'conformation' is meant.
2. lines 186-189 might need a rewrite as wording unclear
>We have also addressed these minor comments.
Reviewer 2:
The revised version of the manuscript incorporate many new and important aspects, discussions and corrections. I believe this article reports and discuss important observations on asymmetric flagellar wave generation and I am confident that the community will appreciate this tremendously. Congratulations to all authors for this beautiful study.
>I would again like to thank Reviewer 2 for their considered review of this work and their kind support.

Third decision letter

MS ID#: JOCES/2020/246637

MS TITLE: The single flagellum of Leishmania has a fixed polarisation of its asymmetric beat

AUTHORS: Ziyin Wang, Tom Beneke, Eva Gluenz, and Richard Wheeler
ARTICLE TYPE: Research Article

I am happy to tell you that your manuscript has been accepted for publication in Journal of Cell Science, pending standard ethics checks. Thank you for your considered additional revisions.

© 2020. Published by The Company of Biologists under the terms of the Creative Commons Attribution License (https://creativecommons.org/licenses/by/4.0/).
